# Design and Simulation of the Biomechanics of Multi-Layered Composite Poly(Vinyl Alcohol) Coronary Artery Grafts

**DOI:** 10.3389/fcvm.2022.883179

**Published:** 2022-06-24

**Authors:** Katie L. Fegan, Naomi C. Green, Melanie M. Britton, Asif J. Iqbal, Lauren E. J. Thomas-Seale

**Affiliations:** ^1^Physical Sciences for Health Centre for Doctoral Training, University of Birmingham, Birmingham, United Kingdom; ^2^Department of Mechanical Engineering, University of Birmingham, Birmingham, United Kingdom; ^3^School of Chemistry, College of Engineering and Physical Sciences, University of Birmingham, Birmingham, United Kingdom; ^4^Institute of Cardiovascular Sciences, University of Birmingham, Birmingham, United Kingdom

**Keywords:** tri-layer graft, finite element analysis (FEA), PVA/gelatin, cryogel, transmural stress distribution, transmural strain distribution, hyperelasticity, cardiovascular tissue engineering

## Abstract

Coronary artery disease is among the primary causes of death worldwide. While synthetic grafts allow replacement of diseased tissue, mismatched mechanical properties between graft and native tissue remains a major cause of graft failure. Multi-layered grafts could overcome these mechanical incompatibilities by mimicking the structural heterogeneity of the artery wall. However, the layer-specific biomechanics of synthetic grafts under physiological conditions and their impact on endothelial function is often overlooked and/or poorly understood. In this study, the transmural biomechanics of four synthetic graft designs were simulated under physiological pressure, relative to the coronary artery wall, using finite element analysis. Using poly(vinyl alcohol) (PVA)/gelatin cryogel as the representative biomaterial, the following conclusions are drawn: (I) the maximum circumferential stress occurs at the luminal surface of both the grafts and the artery; (II) circumferential stress varies discontinuously across the media and adventitia, and is influenced by the stiffness of the adventitia; (III) unlike native tissue, PVA/gelatin does not exhibit strain stiffening below diastolic pressure; and (IV) for both PVA/gelatin and native tissue, the magnitude of stress and strain distribution is heavily dependent on the constitutive models used to model material hyperelasticity. While these results build on the current literature surrounding PVA-based arterial grafts, the proposed method has exciting potential toward the wider design of multi-layer scaffolds. Such finite element analyses could help guide the future validation of multi-layered grafts for the treatment of coronary artery disease.

## Introduction

Cardiovascular disease is the number one cause of death worldwide. In the United Kingdom, coronary artery disease alone killed approximately 60,000 people in 2019 and continues to cost an estimated €9 billion per annum (1). Designing synthetic arterial grafts to replace diseased blood vessels therefore remains a critical field of biomedical research (2–4).

For a graft to remain viable *in vivo*, it must replicate the native function of the artery it aims to replace. This relies on the intrinsic characteristics of the biomaterial(s) used (5). These characteristics are often grouped into four categories: (I) the ability to maintain a healthy monolayer of endothelial cells (an endothelium) on the biomaterial surface; (II) blood compatibility; (III) biocompatibility and biodegradability; and (IV) matched mechanical properties (6). Importantly, the success of a graft depends on satisfying all four categories simultaneously. A healthy endothelium promotes a quiescent non-thrombogenic phenotype to prevent blood clot formation; this phenotype is mediated in part by the biomechanical forces exerted on the endothelium. Arteries expand and contract in response to blood pressure, generating stress and strain within the vessel wall (7). Circumferential (tensile) stretch activates downstream signalling pathways through mechanotransduction, influencing EC phenotype and morphology (8). The extent of stress and strain is determined by the wall stiffness, which in turn influences vessel compliance, defined as the change in arterial volume relative to the change in pressure. Compliance impacts blood flow through the vessel and subsequently the frictional force (shear stress) exerted at the blood—endothelium interface (9, 10). Thus, the biomechanics of the graft need to replicate that of *in vivo* arterial tissue in order to support healthy endothelial function.

While a vast array of vascular mimicking materials (VMMs) have been developed over the last few decades, few have been characterised with the constitutive equations needed to capture the complex mechanical behaviour of the arterial wall (11). Arteries are hyperelastic, viscoelastic and anisotropic (12–14). Their response to loading under pulsatile blood flow is strain-dependent and direction-dependent; at large strain, the stiffness not only increases, but is higher along the longitudinal direction of the vessel. This behaviour is dictated by the arrangement of stiff (collagen) and elastic (elastin) fibrils in the three layers of the arterial wall. Each layer fulfils a specific task. As the luminal layer, the intima supports the endothelium. It is generally thought that the media is responsible for arterial wall properties at physiological pressure (15–17). However, studies in aged human coronary arteries suggest that the intima bears more load in arteries with non-atherosclerotic intimal thickening (18). Meanwhile, the outermost layer, the adventitia, prevents overdistension.

It is reasonable to assume that a single-layer vascular graft could not sufficiently capture all of the complex properties of the artery wall. Instead, multi-layered grafts could overcome the mechanical simplicity of homogeneous VMMs by mimicking the structure–function relationship of the individual layers (19). Tri-layer arterial grafts have been tested *in vitro* (20) and *in vivo* (21–23). Biomimetic vascular scaffolds with enhanced tensile strength and burst pressure have been obtained by supporting porous medial layers with polycaprolactone (21) and polyurethane (PU) (22), adventitia mimicking materials with high ultimate stresses. Meanwhile, by changing the thickness of the medial layer of triple-layered PU scaffolds, the compliance can be tailored to match that of sheep carotid artery (23). *In silico* studies of multi-layer grafts are far less common, despite it being faster and cheaper to predict the mechanical behaviour of different graft designs using computational simulations over physical prototypes. Such studies would provide insight into the transmural biomechanics of multi-layered grafts under physiological loads.

Poly(vinyl alcohol) (PVA) hydrogels are promising VMMs thanks to their biocompatibility and ease of manufacture (24). Specifically, PVA cryogels (physically-crosslinked hydrogels formed *via* one or more freeze–thaw cycles) have gained significant attention in soft tissue engineering as they alleviate the need for cytotoxic crosslinking agents (25, 26). PVA cryogels may even be printed into complex geometries using sub-zero (<0°C) additive manufacturing (27–29). When blended with cell-adhesive macromolecules such as gelatin, the cryogels are conducive to endothelialisation (30–32). Moreover, their high-water content and biphasic nature imparts hyper-viscoelastic properties analogous to soft tissue. The extent of hyperelasticity (33) and viscoelasticity (34) can be tuned by varying the formulation and processing parameters, including the number and duration of freeze–thaw cycles and polymer concentration. These factors make PVA-based cryogels appealing targets for multi-layered arterial grafts.

This study aims to simulate the quasi-static biomechanical response of a hyperelastic, tri-layered finite element (FE) model of a coronary artery graft, informed from the experimental characterisation of PVA/gelatin cryogel. These grafts could be easily manufactured using sub-zero additive manufacturing or casting techniques. The transmural stress and strain distribution and compliance of the synthetic grafts under physiological pressure is validated against a control experiment replicating the native arterial wall. In addition, the impact of three different hyperelastic constitutive models on the simulated biomechanics of the coronary artery will be explored. This study not only looks to expand the current literature surrounding PVA-based arterial grafts, but also presents a computational method that complements the experimental characterisation of hyperelastic VMMs for soft tissue engineering. In both cases, it is anticipated that FE modelling will help guide the future design, manufacture and validation of tri-layered graft constructs for the treatment of coronary artery disease.

## Materials and Methods

### Overview

**FIGURE 1:**
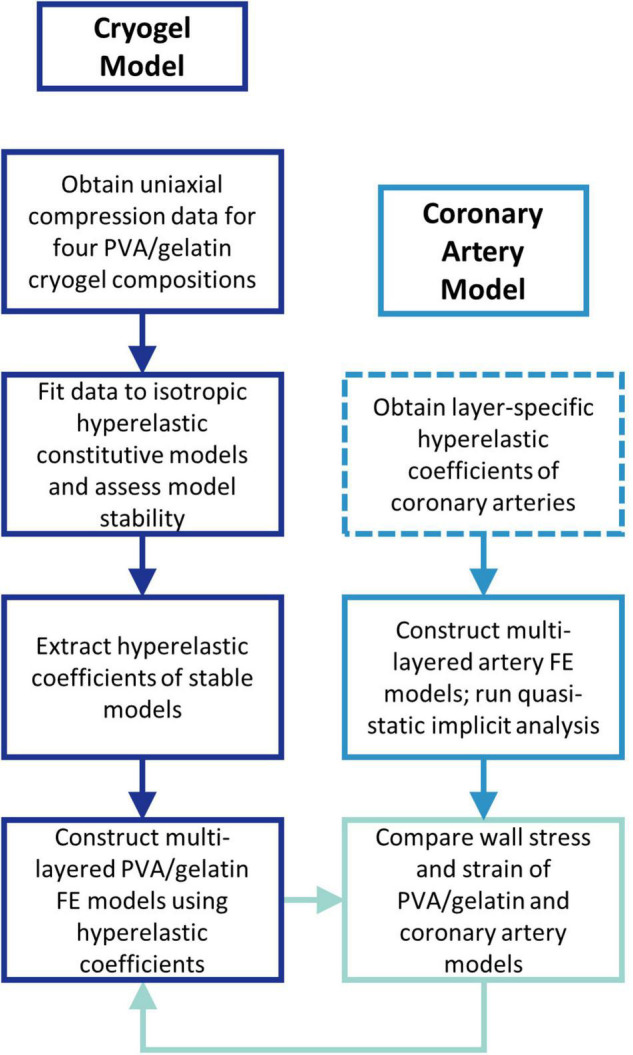
Flow chart outlining the study methodology. Dashed lines represent the steps performed outside of the study.

An overview of the study is given in Figure 1. First, the hyperelastic behaviour of four PVA/gelatin cryogel compositions was determined using uniaxial compression testing. The test data were fitted to a number of isotropic hyperelastic constitutive models; the fit and stability of each model were assessed and the hyperelastic coefficients of stable models were extracted for FE analysis.

A control experiment replicating the native coronary artery wall was performed to provide a benchmark for PVA/gelatin graft behaviour under physiological load. The layer-specific hyperelastic behaviour of human coronary arteries with non-atherosclerotic intimal thickening has been determined experimentally by Holzapfel et al. (18). This data has since been fitted to polynomial (35), reduced polynomial (36) and Ogden (37) models for the simulation of coronary artery biomechanics. However, the rationale behind choosing a specific hyperelastic model to describe the wall layers is not commonly reported. To reflect the variety of hyperelastic models used in the literature, and to investigate the resulting differences in transmural stress and strain between them, three multi-layered FE models of the artery wall, one for each constitutive model, were constructed accordingly. The wall stress and strain distribution of four multi-layered PVA/gelatin constructs were compared to those of the three artery models.

### Chemicals and Materials

PVA (89–98 kDa and 146–186 kDa), gelatin (Type B from bovine skin) and sodium sulphate (Na_2_SO_4_) were acquired from Merck Life Science (Dorset, United Kingdom). Potassium hydroxide (KOH, 85%) was acquired from Alfa Aesar (Lancashire, United Kingdom). Deionised water from a Purite Millipore tank (<18 MΩ, Milli-Q) was used for all experiments unless otherwise stated.

### Preparation of PVA/Gelatin Cryogels

10 wt% PVA/gelatin cryogels were prepared according to the compositions outlined in Table 1. The following nomenclature is used: P_9_G_1_ denotes a weight/weight (w/w) ratio of PVA:gelatin of 9:1; A/B denotes the molecular weight (*M*_*w*_) of PVA (A = 146–186 kDa, B = 89–98 kDa); and C/NC denotes coagulation (C) versus no coagulation (NC) treatment. For example, P_9_G_1_-A-C represents a 10 wt% cryogel with a PVA:gelatin ratio of 9:1, comprised of 146–186 kDa PVA, that has been put through coagulation treatment.

**TABLE 1:** PVA/gelatin cryogel compositions.

Composition	*M*_*w*_ PVA (kDa)	Coagulation treatment
P_9_G_1_-A-NC	146–186	No
P_9_G_1_-A-C	146–186	Yes
P_9_G_1_-B-NC	89–98	No
P_9_G_1_-B-C	89–98	Yes

*P_9_G_1_ signifies a w/w ratio of PVA:gelatin of 9:1, A/B denotes the M_w_ PVA (A = 146–186 kDa, B = 89–98 kDa) and C/NC signifies whether coagulation treatment (C) or no coagulation treatment (NC) was employed during manufacture.*

PVA solutions were prepared by autoclaving PVA in Milli-Q for 1 h at 121°C. Meanwhile, gelatin was dissolved in water at 50°C under magnetic stirring for 1 h. Composite PVA/gelatin solutions were obtained by combining 18 wt% PVA and 2 wt% gelatin in a 1:1 ratio. To prevent aggregation of the polymer, the final solution was mixed at 50°C under constant mechanical stirring for 1 h followed by 1 h at room temperature (22.5 ± 1°C). The solution was then poured into 20 mm × 10 mm cylindrical molds and subjected to three freeze–thaw cycles at −20°C and room temperature respectively.

Once formed, half of the cryogels were immersed in a non-solvent (1 M KOH/1 M Na_2_SO_4_, “coagulation treatment”) for 1 h to promote further crosslinking of PVA. By exchanging bulk water with the non-solvent, amorphous PVA is precipitated out and the polymer microstructure is reinforced. Na_2_SO_4_ further aids PVA precipitation by decreasing the solubility of the polymer, a phenomenon known as the Hofmeister effect (38, 39). Meanwhile, the other half were immersed in water. All cryogels were stored in Milli-Q for 3–4 days to ensure equilibrium swelling was reached (30).

### Uniaxial Compression Testing

PVA/gelatin cryogels were compressively loaded to a maximum of 50% strain at a strain rate of 0.1 mm s^–1^ using a Bose Electroforce 3200 testing machine (Bose Corporation, ElectroForce Systems Group, MN, United States; now TA Instruments, Delaware, United States) equipped with a 220 N load cell. Each sample was subjected to 9 preconditioning cycles to account for stress softening of the polymer chains (40); the force–displacement data were then recorded on the 10th cycle. The data were converted to engineering stress (σ) and engineering strain (ϵ) using Equations 1 and 2, where *A* is the cross-sectional area of the cryogel (measured using a Vernier calliper) and *L_0_* is the initial height of the sample.


(1) $$\sigma =\frac{F}{A}$$

(2) $$\epsilon =\frac{△\,L}{L_{0}}$$

In this study, all compressive stresses and strains are presented as their absolute values. The resulting stress–strain curves contained inherent noise due to the resolution of the load cell. Noise reduces the quality of the hyperelastic constitutive modelling process described in section “Material Properties”: therefore, prior to this analysis, it was imperative to smooth the data such that stress varied smoothly with strain. All data were smoothed by sampling stress–strain pairs in intervals of 40 between 0 and 25% strain. An interval of 20 was used between 25 and 50% strain to better capture the stiffening behaviour.

### Density Measurements

The density of each PVA/gelatin composition was measured in preparation for the FE model. A geometric approach was used to relate the volume, *V*, of each cylindrical sample to its mass, *m* (Equation 3). The specimens were dried of surface water immediately before their masses were recorded using a mass balance.


(3) $$\rho =\frac{m}{v}=\frac{4\,m}{\pi \,d^{2}\,L}$$

### Finite Element Model

**FIGURE 2:**
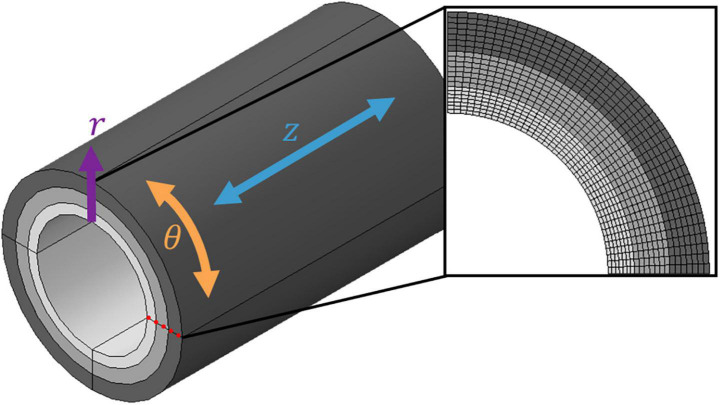
Discretised three-layer coronary artery wall. A cylindrical coordinate system was adopted using the three-dimensional coordinates **r** (radial), θ (circumferential) and **z** (axial). Each layer was meshed with seven layers of elements through the radial thickness; the nodal analysis path used to extract field variable values from the model is highlighted by the red dotted line.

All finite element (FE) models were constructed in Abaqus/CAE 2021 (Dassault Systèmes, SIMULIA Corp., Johnston, RI, United States) using a quasi-static implicit analysis. The results of transmural stress and strain were taken as nodal analysis paths through the thickness of the wall, as shown in Figure 2.

#### Geometry

The coronary artery was idealised as a straight cylindrical vessel partitioned into three concentric tissue layers. The thicknesses of the intima, media, and adventitia were chosen according to experimental data on human coronary arteries (18) (0.23, 0.30 and 0.34 mm, respectively). The artery was then embedded within a representative block of soft tissue to represent the surrounding connective tissues.

#### Material Properties

The test data were fit to a number of phenomenological isotropic hyperelastic constitutive models using Abaqus’ non-linear regression routine. These models use strain energy density functions, *U*, to relate the amount of energy needed to deform a unit volume of a material to the imposed strain at that volume. In this study, variations of the polynomial (P) strain energy function were investigated:


(4) $$U_{\text{P}}=\sum_{i+j=1}^{N}C_{i\,j}\,{\left({\bar{I}}_{1}-3\right)}^{i}\,{\left({\bar{I}}_{2}-3\right)}^{j}+\sum_{i=1}^{N}\frac{1}{D_{i}}\,{\left(J_{e\,l}-1\right)}^{2\,i}$$

where *U* is the strain energy per unit volume; *N* is the order of the polynomial; ${\bar{I}}_{1}$ and ${\bar{I}}_{2}$ are the first and second invariants of the deviatoric Cauchy–Green tensor; *J*_*el*_ is the elastic volume ratio; and *C*_*ij*_ and *D_i_* are material constants that describe the shear (*C*_*ij*_) and compressibility (*D_i_*) behaviour of the material. As PVA is considered near-incompressible (41), *D*_*i*_=0 and the compressibility term is subsequently eliminated:


(5) $$U_{\text{P}}=\sum_{i+j=1}^{N}C_{i\,j}\,{\left({\bar{I}}_{1}-3\right)}^{i}\,{\left({\bar{I}}_{2}-3\right)}^{j}$$

If the second invariant of deviatoric strain is omitted, the polynomial model degenerates into a reduced polynomial (RP) model of the general form:


(6) $$U_{\text{RP}}=\sum_{i=1}^{N}C_{i\,0}\,{\left({\bar{I}}_{1}-3\right)}^{i}$$

Specific forms of each model are obtained for specific choices of *C*_*ij*_. For example, the Mooney-Rivlin form follows the 1*^st^* order polynomial model:


(7) $$U_{\mathrm{Mooney}-\mathrm{Rivlin}}=C_{10}\,\left({\bar{I}}_{1}-3\right)+C_{01}\,\left({\bar{I}}_{2}-3\right)$$

Removing the second deviatoric strain invariant from the Mooney-Rivlin form yields the 1*^st^* order reduced polynomial, or neo-Hookean, form. Meanwhile, when *N* = 3, the Yeoh form is obtained:


(8) $$U_{\mathrm{neo}-\mathrm{Hookean}}=C_{10}\,\left({\bar{I}}_{1}-3\right)$$

(9) $$U_{\text{Yeoh}}=C_{10}\,\left({\bar{I}}_{1}-3\right)+C_{20}\,{\left({\bar{I}}_{1}-3\right)}^{2}+C_{30}\,{\left({\bar{I}}_{1}-3\right)}^{3}$$

By contrast, the Ogden model is a specialised form of the polynomial model that is expressed by principle stretches instead of Cauchy–Green invariants:


(10) $$U_{\text{Ogden}}=\sum_{i=1}^{N}\frac{2\,\mu_{i}}{\alpha_{i}^{2}}\,\left({\bar{\lambda }}_{1}^{\alpha_{i}}+{\bar{\lambda }}_{2}^{\alpha_{i}}+{\bar{\lambda }}_{3}^{\alpha_{i}}-3\right)$$

where ${\bar{\lambda }}_{i}$ are the principle stretch ratios; and μ_*i*_ and α_*i*_ material constants describing the shear behaviour. The parameters of each model were fitted to the test data using a least squares optimisation procedure. The stability of each hyperelastic model was then assessed using the Drucker stability criterion, which states that stress must continuously increase for increasing strain (and vice versa) (42). Unstable models were discounted from further analyses. The correlation between the test data and each stable hyperelastic model, or “goodness of fit”, was established using Pearson’s correlation coefficient, *r*^2^:


(11) $$r^{2}={\left(\frac{\sum_{i=1}^{N}\left(\sigma_{i,\exp}-{\bar{\sigma }}_{\text{exp}}\right)\,\left(\sigma_{i,\mathrm{model}}-{\bar{\sigma }}_{\text{model}}\right)}{\sqrt{\sum_{i=1}^{N}{\left(\sigma_{i,\exp}-{\bar{\sigma }}_{\text{exp}}\right)}^{2}\,\sum_{i=1}^{N}{\left(\sigma_{i,\mathrm{model}}-{\bar{\sigma }}_{\text{model}}\right)}^{2}}}\right)}^{2}$$

where, for *N* stress–strain pairs, σ_*i*,*exp*_ is the experimental stress and σ_*i*,*model*_ is the stress derived from the stress–strain relation of each strain energy function. Derivation of these stress–strain relations is described in detail elsewhere (43). The hyperelastic parameters for each cryogel composition were averaged to provide single material constants for FE analysis (see Data Analysis). A displacement-driven uniaxial compression FE model matching the experimental setup was simulated using these constants and the resulting stress–strain responses were compared to the individual test data.

To allow direct comparison of PVA/gelatin cryogel and coronary artery hyperelastic parameters, a common parameter that can be derived from all constitutive models was needed. The initial shear modulus, μ_0_, was calculated using Equations 12–14.


(12) $$\mu_{0,P}=2\,\left(C_{10}+C_{01}\right)$$

(13) $$\mu_{0,\mathrm{RP}}=2\,\left(C_{10}\right)$$

(14) $$\mu_{0,\mathrm{Ogden}}=\frac{1}{2}\,\sum_{i=1}^{N}\mu_{i}\,\alpha_{i}$$

Layer-specific hyperelastic coefficients of human coronary arteries were obtained from the literature and are listed in Table 2. The surrounding soft tissue was modelled as a Hookean linearly elastic material with a Young’s modulus of 0.05 MPa (44) and a Poisson’s ratio of 0.49. The tissue layers were modelled using an average value of density for soft tissue (1.04 ×10^–6^ kg mm^–3^) according to a previous study (44).

**TABLE 2:** Layer-specific hyperelastic material coefficients derived from hyperelastic isotropic constitutive models fitted to coronary arterial tissue.

	Material parameters			
Material model	Intima	Media	Adventitia	From study
Polynomial	*C*_10_=−0.204 MPa, *C*_01_ = 0.223 MPa, *C*_20_=1.37 MPa, *C*_11_ = −3.71 MPa, *C*_02_ = 2.67 MPa	*C*_10_=−0.117 MPa, *C*_01_ = 0.128 MPa, *C*_20_=0.224 MPa, *C*_11_ = -0.672 MPa, *C*_02_ = 0.569 MPa	*C*_10_=−0.189 MPa, *C*_01_ = 0.202 MPa, *C*_20_=0.459 MPa, *C*_11_ = −1.38 MPa, *C*_02_ = 1.34 MPa	(35)
Reduced Polynomial	*C*_10_=6.79 ×10^–3^ MPa, *C*_20_ = 0.54 MPa, *C*_30_=−1.11 MPa, *C*_40_=10.65 MPa, *C*_50_=−7.27 MPa, *C*_60_ = 1.63 MPa	*C*_10_ = 6.52 ×10^–3^ MPa, *C*_20_ = 4.89 ×10^–2^ MPa, *C*_30_=9.26 ×10^–3^ MPa, *C*_40_=0.76 MPa, *C*_50_=−0.43 MPa, *C*_60_ = 8.69 ×10^–2^ MPa	*C*_10_=8.27 ×10^–3^ MPa, *C*_20_=1.20 ×10^–2^ MPa, *C*_30_=0.52 MPa, *C*_40_=−5.63 MPa, *C*_50_ = 21.44 MPa, *C*_60_ = 0.00 MPa	(36)
Ogden	μ_1_ = −5.70 MPa μ_2_=3.58 MPa μ_3_ = 2.17 MPa α_1_ = 24.43 α_2_ = 25.00 α_3_ = 23.24 *D*_1_=0.85	μ_1_ = −1.84 MPa μ_2_=1.12 MPa μ_3_ = 0.73 MPa α_1_ = 21.71 α_2_ = 22.00 α_3_ = 21.20 *D*_1_=4.11	μ_1_ = −1.99 MPa μ_2_=1.20 MPa μ_3_ = 0.81 MPa α_1_ = 24.61 α_2_ = 25.00 α_3_ = 23.90 *D*_1_=3.92	(37)

#### Boundary Conditions

Using radial (*r*), circumferential (θ) and axial (*z*) cylindrical coordinates (Figure 2), the outer surface of the soft tissue was constrained circumferentially in all six degrees of freedom. The inlets of the soft tissue and each tissue layer were also constrained axially. This allowed the artery to expand freely in both the radial and circumferential directions. The load was applied radially to the luminal surface of the model using two analysis steps: an 80 mmHg pressure load was first applied to represent diastolic blood pressure, and an additional 40 mmHg was applied to reach a systolic blood pressure of 120 mmHg.

To replicate the behaviour between heterogeneous tissue and material boundaries, as opposed to a homogeneous construct, the following interface constraints were applied. The contact between each tissue layer was modelled using surface-to-surface interactions with hard penalty contact constraints. Under the quasi-static luminal pressure applied in this study, assuming these hard penalty contact constraints, there will be no relative radial motion between the interfacing surfaces. To enable circumferential motion yet also prevent unconstrained sliding, static friction coefficients were used to model the behaviour between interfacing arterial tissue and PVA/gelatin layers. There is very limited research exploring the interactions between the surfaces of the artery wall layers and the surfaces of hydrogels. In their balloon angioplasty analysis, Mortier et al. (45) applied a friction coefficient of 0.2 between all contact pairs. While the friction coefficient between interfacing PVA layers has not been reported, the biotribological behaviour of PVA and titanium alloy yielded a friction coefficient of 0.04 (46). These estimated values were applied in this study.

#### Mesh

The artery and surrounding soft tissue were discretised using solid linear hexahedral (C3D8RH) elements. Hybrid (H) elements were used to prevent volumetric locking, while reduced integration (R) elements were chosen to account for geometric nonlinearity. A mesh convergence study was conducted to ensure the values of stress obtained were independent of the mesh (Supplementary Figure 1). The resulting mesh contained a total of 171,200 elements, with an edge length of 0.36 mm, for the wall layers. Each layer contained seven layers of elements through the radial thickness (Figure 2).

### Data Analysis

A total of eight samples were tested per cryogel composition. All constitutive parameters are presented as mean ± standard deviation (SD). Although single sets of optimal Ogden constitutive parameters were obtained for three of the compositions, two sets were obtained for P_9_G_1_-B-C: for one set, α_1_ > 0, and for the other, α_1_ < 0. Both sets are equally valid combinations of coefficients and yielded similar *r*^2^ values (Supplementary Table 1). To allow averaging of the Ogden coefficients, samples where α_1_ < 0 were excluded from the dataset. The sample size was subsequently reduced to *n* = 6 for all PVA/gelatin compositions and for the calculation of mean ±SD of all other stable constitutive models. The effect of *M*_*w*_ and coagulation treatment on *C*_*ij*_ and μ_0_of PVA/gelatin cryogels were analysed in SigmaPlot 14.5 (Systat Software Inc, San Jose, CA, United States) using one-way ANOVA with the Holm-Sidak post-hoc test.

## Results

### Hyperelastic Constitutive Modelling of PVA/Gelatin

When compressed to large (50%) compressive strains, each PVA/gelatin composition exhibited a characteristic “J”-shaped hyperelastic stress–strain response to loading. The stress–strain curves for individual samples are included in the Supplementary Material (Supplementary Figure 2). All phenomenological hyperelastic isotropic models routinely available in Abaqus were fitted to the test data. Those satisfying the Drucker stability criterion under uniaxial, biaxial and shear deformation, across all strain ranges, were then checked for accuracy according to their *r*^2^ value.

**FIGURE 3:**
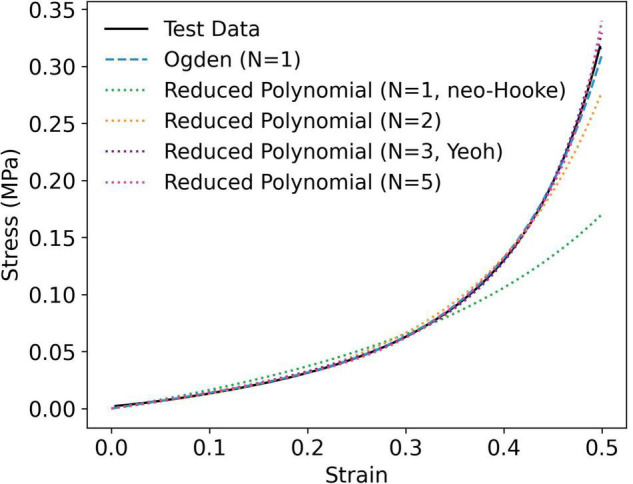
Representative curve fit of stable hyperelastic models to uniaxial compression data, taken from an individual P_9_G_1_-A-C sample.

While the Mooney-Rivlin model provided a stable fit for some samples, it yielded instabilities in others (Supplementary Table 2). The neo-Hookean model, on the other hand, was unconditionally stable, as were the first order Ogden and second, third (Yeoh) and fifth order reduced polynomial models (Figure 3 and Supplementary Table 3). The correlations between each model and the test data were close to unity (1 < *r*^2^ > 0.9). Nevertheless, the neo-Hookean model underestimated stress at large strains, yielding the weakest correlation for all PVA/gelatin compositions (*r*^2^≈ 0.93). The approximation of stress at higher strains was improved by increasing the number of terms in the reduced polynomial model, as evidenced by increasing *r*^2^ values; however, similar *r*^2^values were observed between Yeoh and fifth order reduced polynomial models (*r*^2^≈0.99). Meanwhile, a single-term Ogden model was sufficient to capture the strain-stiffening behaviour of PVA/gelatin cryogel (*r*^2^ > 0.99). The average Ogden and Yeoh constitutive parameters for the stable models are presented in Table 3.

**TABLE 3:** First order Ogden and Yeoh constitutive parameters fitted to PVA/gelatin compression data and densities of all PVA/gelatin cryogel compositions (mean ± SD).

	First order Ogden constitutive parameters			
	P9G1-A-C	P9G1-A-NC	P9G1-B-C	P9G1-B-NC
μ_1_ (MPa)	0.0444 ± 0.0060	0.0374 ± 0.0042	0.0276 ± 0.0010	0.0227 ± 0.0025
α_1_	7.116 ± 0.528	6.553 ± 0.276	7.765 ± 0.493	7.311 ± 0.581
μ_0_ (MPa)	0.157 ± 0.016	0.122 ± 0.012	0.107 ± 0.009	0.083 ± 0.011
*r* ^2^	0.9994 ± 0.0004	0.9988 ± 0.0008	0.9994 ± 0.0003	0.9988 ± 0.0008

	**Yeoh constitutive parameters**			
	
	**P9G1-A-C**	**P9G1-A-NC**	**P9G1-B-C**	**P9G1-B-NC**

*C*_10_ (MPa)	0.0205 ± 0.0035	0.0180 ± 0.0024	0.0126 ± 0.0006	0.0108 ± 0.0014
*C*_20_ (MPa)	0.0015 ± 0.0028	−0.0015 ± 0.0019	0.0015 ± 0.0014	−0.0007 ± 0.0007
*C*_30_ (MPa)	0.0042 ± 0.0017	0.0044 ± 0.0012	0.0032 ± 0.0006	0.0033 ± 0.0009
μ_0_ (MPa)	0.0410 ± 0.0070	0.0360 ± 0.0049	0.0252 ± 0.0012	0.0217 ± 0.0027
*r* ^2^	0.9994 ± 0.0008	0.9984 ± 0.0009	0.9997 ± 0.0002	0.9985 ± 0.0007
**Density**				
ρ(*kgm*^3^)	1080 ± 36	1190 ± 19	1070 ± 37	1120 ± 38

Statistical analysis of the reduced polynomial parameter *C*_*10*_, interpreted as $\frac{1}{2}\,\mu_{0}$, revealed a significant increase in stiffness as a function of *M_w_* PVA for the Yeoh and fifth order polynomial models (*p* < 0.05), but not as a result of coagulation treatment (*p* > 0.05, Supplementary Table 4). Conversely, a statistically significant increase in the Ogden parameter μ_1_ was observed as a function of *M*_*w*_ PVA or use of coagulation treatment. The same trend was observed for μ_0_. For all compositions, |α_1_| > 2. This reflects the stiffening effect observed at higher strain (47). However, the only statistically significant change in α_1_ when varying the manufacturing parameters was between P_9_G_1_-A-NC and P_9_G_1_-B-C (*p* = 0.02).

### Validation of Yeoh and Ogden Constitutive Parameters for FE Modelling

**FIGURE 4:**
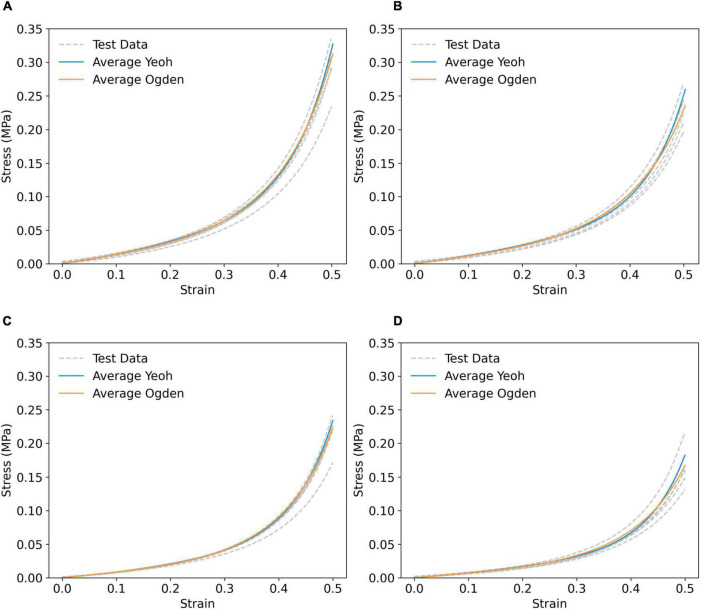
Simulated compressive stress–strain response using averaged Yeoh (solid blue line) and first order Ogden (solid orange) parameters of **(A)** P_9_G_1_-A-C, **(B)** P_9_G_1_-A-NC, **(C)** P_9_G_1_-B-C, and **(D)** P_9_G_1_-B-NC, compared to experimental test data (grey dashed lines).

The Yeoh and first order Ogden models were taken forward to represent the hyperelastic behaviour of PVA/gelatin cryogel in all FE models. The mean stress–strain responses, yielded by the mean values of each model’s constitutive parameters, were obtained using a displacement-driven uniaxial compression FE model and compared to the experimental stress–strain responses of individual samples (Figure 4). This method successfully generated master curves reflecting the mean mechanical properties of each cryogel composition, with strain-stiffening behaviour analogous to the test data.

### Comparison of Coronary Artery Model Transmural Biomechanics

**FIGURE 5:**
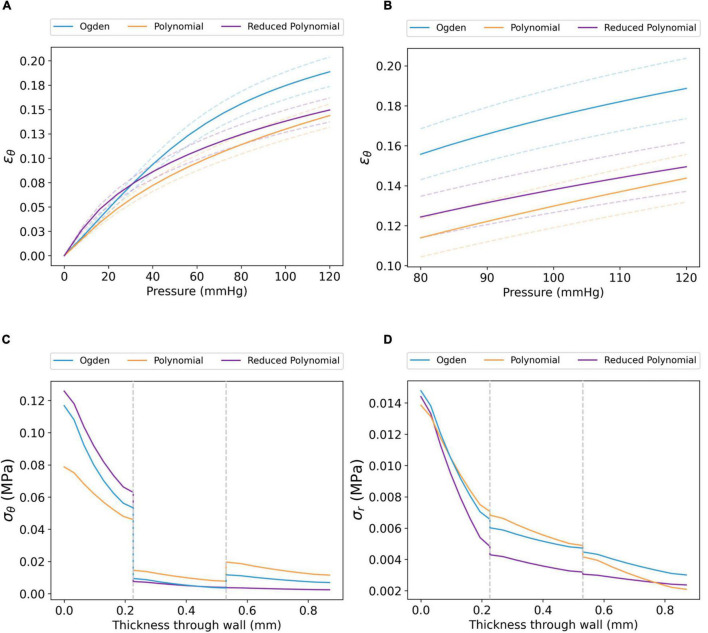
Transmural biomechanics of the coronary artery using the Ogden, polynomial and reduced polynomial hyperelastic parameters listed in Table 2. The mean ϵ_θ_ of the coronary intima between **(A)** 0–120 and **(B)** 80–120 mmHg pressure (dashed lines show the S.D. for each model). Similar trends were observed for ϵ_*θ,media*_, ϵ_*θ,adventitia*_, ϵ_*r,intima*_, ϵ_*r,media*_ and ϵ_*r,adventitia*_; for these, the reader is referred to Supplementary Figures 3–5. **(C)** σ_θ_ and **(D)** σ_*r*_ profiles for each model through the radial thickness of the artery wall, extracted at systolic pressure. Grey dashed lines represent the intima–media and media–adventitia interfaces.

The tri-layer FE model was first run using layer-specific hyperelastic material coefficients fitted to coronary arterial tissue (Table 2). The strain response of each layer of the artery model showed a non-linear relationship with pressure across the 0–120 mmHg pressure range. Demonstrated for the circumferential strain (ϵ_θ_) of the intima in Figures 5A,B, this indicates a strain-stiffening response at higher physiological pressures, a phenomenon well documented experimentally (48, 49). Analogous behaviour was observed for ϵ_*r,intima*_ (Supplementary Figure 3). To prevent repetition, ϵ_*θ,media*_/ϵ_*r,media*_ and ϵ_*θ,adventitia*_/ϵ_*r,adventitia*_ are included in Supplementary Figures 4, 5. The response was approximately linear between diastole (80 mmHg) and systole (120 mmHg). Furthermore, the difference in ϵ_θ_ between each model was largest at systolic pressure: for example, ϵ_θ,*intima*_=0.14 ±0.01, 0.15 ±0.01 and 0.19 ±0.02 MPa in the polynomial, reduced polynomial and Ogden models, respectively. A similar trend was observed for ϵ_*r*_ (ϵ_*r,intima*_ = 0.13 ±0.01, 0.13 ±0.01 and 0.15 ±0.01 MPa, respectively).

The circumferential stress (σ_θ_) and radial stress (σ_*r*_) at systolic pressure, as a function of radial wall thickness, are displayed in Figures 5C,D. For all three coronary artery models, the distribution of σ_θ_ was non-uniform across the three tissue layers (Figure 5C). Each model demonstrated maximum σ_θ_ at the luminal surface, followed by a marked decrease at the intima–media interface. Of the three models, the maximum value of σ_θ_ was lower in the polynomial model (σ_θ_=0.079 MPa) than in the Ogden (σ_θ_=0.117 MPa) and reduced polynomial (σ_θ_=0.126 MPa) models. Though a further increase in σ_θ_ was observed between the media and adventitia in the Ogden and polynomial models, σ_θ_ distribution transitioned continuously across the media–adventitia interface in the reduced polynomial model. The effects of compressive stresses through the wall cannot be neglected as the artery has a radius-to-thickness ratio of less than 10, necessitating the thick-walled assumption. In all cases, σ_*r*_ was an order of magnitude lower than σ_θ_ and decreased with increasing distance from the luminal surface (Figure 5D).

### PVA/Gelatin Graft Design Validation

Based on the transmural trends observed from the coronary artery models in section “Comparison of Coronary Artery Model Transmural Biomechanics,” the following targets were set for the PVA/gelatin graft constructs:

(I) for σ_θ_, σ_θ,*intima*_ > σ_θ,*media*_≤σ_θ,*adventitia*_;
(II) for σ_*r*_, σ_*r*,*intima*_ > σ_*r*,*media*_ > σ_*r*,*adventitia*_;
(III) for ϵ_θ_ and ϵ_*r*_, strain-stiffening should occur between 0 and 120 mmHg pressure.

The original test data of Holzapfel et al. (18) and the results from “Comparison of Coronary Artery Transmural Biomechanics” of this study show that the coronary intima displays substantial mechanical strength and load-bearing properties. By contrast, the media is the softest of the three tissue layers. The intima was therefore set to the stiffest PVA/gelatin composition (P_9_G_1_-A-C), and the media was set to the most compliant PVA/gelatin composition (P_9_G_1_-B-NC), using the Ogden and Yeoh parameters fitted in section “Hyperelastic Constitutive Modelling of PVA/Gelatin.” The adventitia was then varied across the four cryogel compositions tested in the study (Table 4).

**TABLE 4:** PVA/gelatin (PG) compositions used in the four iterations of the PVA/gelatin FE model.

	PG-1	PG-2	PG-3	PG-4
Intima	P_9_G_1_-A-C	P_9_G_1_-A-C	P_9_G_1_-A-C	P_9_G_1_-A-C
Media	P_9_G_1_-B-NC	P_9_G_1_-B-NC	P_9_G_1_-B-NC	P_9_G_1_-B-NC
Adventitia	P_9_G_1_-A-C	P_9_G_1_-A-NC	P_9_G_1_-B-C	P_9_G_1_-B-NC

**FIGURE 6:**
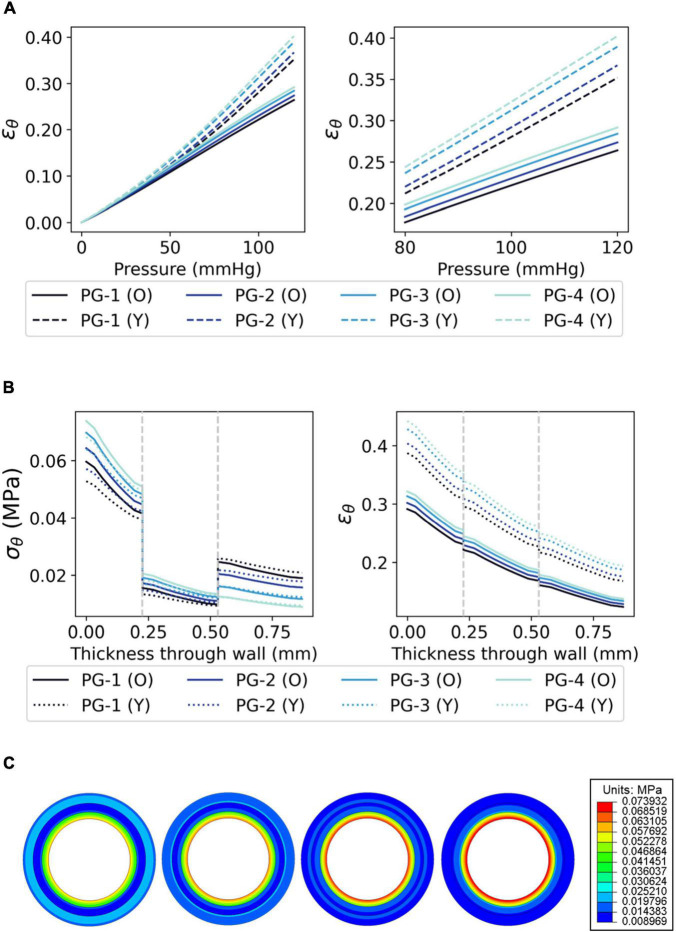
The circumferential biomechanics of PVA/gelatin grafts at systolic pressure. **(A)** The mean ϵ_θ_ of the PVA/gelatin graft intima between 0–120 and 80–120 mmHg pressure (error bars have been omitted for clarity). Similar trends were observed for ϵ_*θ,media*_, ϵ_*θ, adventitia*_, ϵ_*r,intima*_, ϵ_*r,media*_ and ϵ_*r,adventitia*_; for these, the reader is referred to Supplementary Figures 6–8; **(B)** σ_θ_ and ϵ_θ_ profiles through the radial thickness of each PVA/gelatin graft design, modelled using Ogden (solid lines) and Yeoh (dashed lines) hyperelastic parameters. Grey dashed lines represent the intima–media and media–adventitia interfaces; **(C)** From left to right, σ_θ_ contour maps of each PVA/gelatin graft design using Ogden parameters.

**FIGURE 7:**
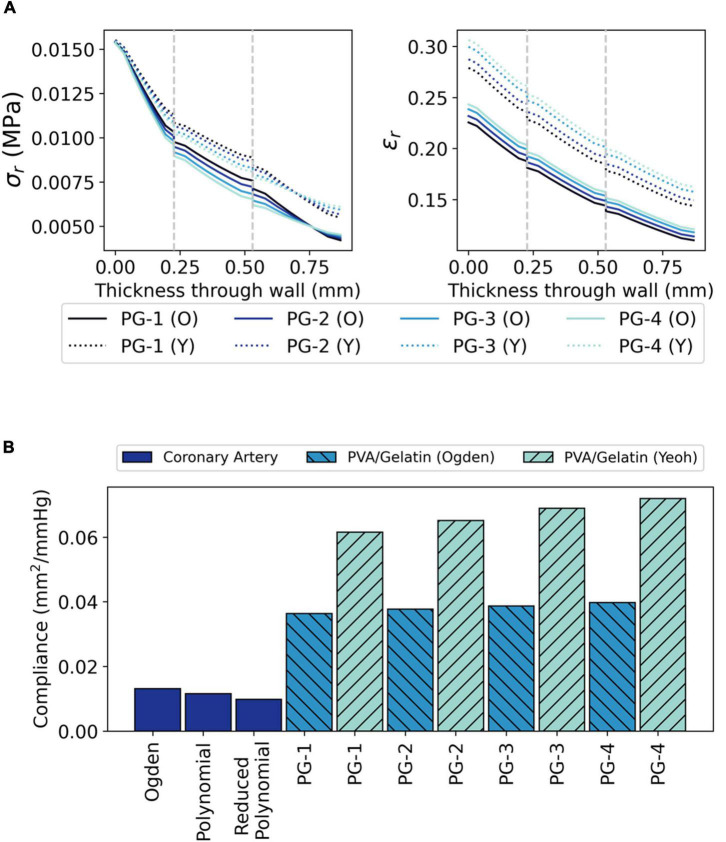
The radial biomechanics of PVA/gelatin grafts at systolic pressure. **(A)** σ_*r*_ and ϵ_*r*_ profiles through the radial thickness of each PVA/gelatin graft modelled using Ogden (solid lines) and Yeoh (dashed lines) hyperelastic parameters. Grey dashed lines represent the intima–media and media–adventitia interfaces; **(B)** Compliance of PVA/gelatin grafts compared to coronary artery models.

The distributions of σ_θ_, ϵ_θ_, σ_*r*_, and ϵ_*r*_ demonstrate a qualitative correlation between the histologically derived coronary artery models (Figure 5) and the synthetic graft designs (Figures 6, 7). Figures 6B,C show the circumferential biomechanics of the four PVA/gelatin graft designs outlined in Table 4 at systolic pressure. σ_θ_ was largest at the luminal surface of all four grafts, comparable to the location of maximum σ_θ_ in the three coronary artery models. Moreover, σ_θ_ decreased across the intima–media interface.

Grafts PG-1, PG-2, and PG-3 replicate the behaviour of a tri-layered system, with an increase in circumferential stiffness provided by the adventitial layer. This mimics the behaviour of the Ogden and polynomial coronary artery models (Figure 5C). The distribution of σ_θ_ was discontinuous at the media–adventitia interface; σ_θ,*adventitia*_ increased with increasing stiffness of adventitia mimicking material (PG-1 > PG-2 > PG-3). Meanwhile, σ_θ,*intima*_ decreased (Figures 6B,C). This suggests that increasing the stiffness of the adventitia shifts the distribution of σ_θ_ from the intima to the adventitia (and, simultaneously, ϵ_θ_ is reduced across all three tissue layers). On the other hand, PG-4 displayed no discernible change in stiffness between the media and adventitia. Instead, PG-4 behaves like a bi-layered graft consisting of a stiff intima and compliant media–adventitia with biomechanics akin to the reduced polynomial coronary artery model. As the graft with the most compliant adventitia mimic, PG-4 exhibited the highest maximum σ_θ_ and ϵ_θ_ of the four designs.

The radial biomechanics of PG-1, PG-2, PG-3 and PG-4 mimicked that of both bi- and tri-layered coronary artery models. At approximately the same magnitude as the applied pressure (120 mmHg ≈ 0.016 MPa), σ_*r*_ was highest at the luminal surface of the graft with stepwise decreases between each tissue layer (Figure 7A).

For each bi- and tri-layered graft and its relevant artery model equivalent, stress and strain were on the same order of magnitude. However, there were some notable location-specific quantitative differences which vary in the degree of error. The relative differences in transmural stress and strain distribution between the synthetic grafts and arterial models (σ_θ,*r*(*diff*)_ and ϵ_*θ,r(diff)*_) at 120 mmHg pressure were calculated using Equation 15 and are plotted in Supplementary Figures 9–11.


(15) $$\sigma ,\epsilon_{\theta ,r\,\left(\mathrm{diff}\right)}=\frac{\sigma ,\epsilon_{\theta ,r\,\left(\mathrm{PVA}/\mathrm{gelatin}\right)}-\sigma ,\epsilon_{\theta ,r\,\left(\mathrm{artery}\right)}}{\sigma ,\epsilon_{\theta ,r\,\left(\mathrm{artery}\right)}}\times 100\%$$

Key discrepancies were observed at the luminal surface of the grafts and the interfaces between tissue layers. These differences are compounded by the choice of hyperelastic model used to model both PVA/gelatin and the coronary artery. Focusing on σ_θ_ distribution, the magnitude of σ_θ_at the luminal surface of PG-1, PG-2 and PG-3 when modelling the cryogels using Ogden parameters was 40–49% lower than that observed in the Ogden coronary artery model and 12–24% lower than that observed in the polynomial coronary artery model. Using Yeoh cryogel parameters, σ_θ(*diff*)_ at the same location were 45–55% and 19–33% lower, respectively.

While σ_θ_ was larger at the luminal surface of the coronary artery than in the PVA/gelatin grafts, the opposite was true at the intima–media interface. Of the tri-layered coronary artery models,σ_θ_ more closely aligned with the polynomial model (7–32% and –8–17% larger when using Ogden and Yeoh cryogel parameters, respectively) than the Ogden model (65–103 and 42–80% larger, respectively). Together, these results indicate that the difference in stiffness between native intima and native media is higher than the PVA/gelatin intima/media mimics used in this study (P_9_G_1_-A-C and P_9_G_1_-B-NC). Indeed, the difference between μ_0,*intima*_ and μ_0,*media*_ in the Ogden coronary artery model and Ogden-based PVA/gelatin grafts was larger in the former (0.255 MPa) than the latter (0.074 MPa).

Unlike the coronary artery, the strain response of each layer of the PVA/gelatin graft showed an approximately linear relationship with pressure across the 0–120 mmHg pressure range (Figure 6A). Strain stiffening was not observed at physiological pressure. ϵ_θ_ and ϵ_*r*_ were subsequently higher in all synthetic grafts than in each coronary artery model at systolic pressure. This result was more pronounced when using the Yeoh model to describe the material response of PVA/gelatin. The compliance, taken here as the relative change in cross-sectional area between diastolic and systolic pressure, is related to ϵ_*r*_ and can be used as a general marker of graft stiffness. For all three arterial models, compliance mismatch was lowest for PG-1 and largest for PG-4 (Figure 7B). The relative error between graft and artery compliance was reduced by using the Ogden model to define the material response of PVA/gelatin. For example, the compliance of PG-1 modelled using Ogden parameters was 177–270% higher than the coronary artery models, compared to 369–527% for the corresponding Yeoh parameters.

## Discussion

Tri-layered grafts are gaining traction in small-diameter blood vessel tissue engineering as a strategy to reduce the mechanical incompatibilities associated with homogeneous grafts (19). FE analysis has the potential to rapidly accelerate the design process for engineering biomimetic synthetic grafts by highlighting the parameters which promote specific mechanical behaviours (50). However, its use in the design and validation of multilayered synthetic arterial grafts is relatively unexplored.

In arteries, circumferential stretch activates various EC mechanoreceptors regulating extracellular matrix remodeling, vascular tone homeostasis and inflammation (8). Understanding the influence of graft stress and strain on EC behaviour in relation to the local stresses of the native artery wall may therefore provide insight on graft performance. Yet, no studies have investigated the transmural stress and strain distribution through multi-layered grafts. This study therefore aimed to evaluate the transmural biomechanics of synthetic PVA/gelatin grafts under quasi-static loading using FE analysis and subsequently compare the stress and strain distributions to coronary artery tissue.

Like arteries, hydrogels are nearly incompressible and demonstrate a non-linear response to loading. Thus, a hyperelastic model was needed to emulate the mechanical behaviour of both the artery and the synthetic graft construct. This study first characterised the hyperelasticity of four PVA/gelatin cryogel compositions according to a number of isotropic models used to empirically describe rubbery materials. Pure PVA cryogels have previously been fitted to first (33, 51) and second (52) order polynomial, Yeoh and Ogden (53) models. Here, the hyperelastic response of PVA/gelatin cryogels has been calibrated to polynomial, reduced polynomial and Ogden models of varying order, using data obtained under uniaxial compression.

For a hyperelastic model to be efficient, it should contain as few terms as possible. This ensures that each coefficient describes some part of the mechanical response of the material and minimises the number of experimental tests needed to fit them (54, 55). The model must also be stable across the strain region of interest. Stability is especially important when characterising a synthetic graft material because the FE model must accurately predict its behaviour when subjected to multi-axial stress states, such as those experienced in a pressurised artery. In this study, the stability of PVA/gelatin cryogel under biaxial and planar shear is considered qualitatively using the material constants obtained under uniaxial compression, as neither biaxial nor shear tests were performed experimentally. This approach has been implemented in the characterisation of other hyperelastic materials (56).

The Mooney-Rivlin model failed to consistently predict the hyperelastic behaviour of each cryogel composition; it yielded Drucker instabilities under uniaxial, biaxial and planar compression and tension. Removing the second strain invariant overcame these instabilities. However, while the neo-Hookean model is suited to limited test data (54), it could not capture the strain hardening effect observed at high (>30%) strain. This is reflected in the correlation coefficient, which was notably lower than the other stable models for all cryogel compositions. Increasing the order of the reduced polynomial improved the approximation of the stiffening response of PVA/gelatin cryogel at high strain. The third order reduced polynomial (Yeoh) model provided the necessary accuracy at high strain while keeping the order of the strain energy density function as low as possible.

The first order Ogden model provided a good fit using only two coefficients: μ_1_, with units of pressure, and α_1_, a dimensionless quantity that determines the non-linearity of the stress–strain plot. Together, they describe the shear modulus of the cryogel. The Ogden model has been used to capture the hyperelastic behaviour of many soft strain-hardening materials, including gelatin gels (57). Second and third order Ogden models need test data from multiple deformation modes for a stable fit; however, other studies have demonstrated that the first order model can properly characterise hydrogels under compression (57, 58).

Analysis of PVA/gelatin Ogden parameters shows that increasing the *M_w_* of PVA or using coagulation treatment increased μ_1_ and thus the stiffness of the cryogel (*p* < 0.05). This agrees with previous literature, where higher elastic (30, 59) and dynamic complex (34) moduli have been linked to changes in the gel microstructure caused by the cryogelation process. No statistical significance in α_1_ was observed as either a function of *M_w_* PVA or use of coagulation treatment. This indicates that the curvature of the stress–strain plot is independent of both manufacturing parameters and each composition exhibits the same overall trend in stress-stiffening response over 50% strain. Interestingly, the Yeoh model did not highlight a statistically significant increase in its stiffness parameter *C*_*10*_ when using coagulation treatment. This discrepancy may be explained by considering that $C_{10}=\frac{1}{2}\,\mu_{0}$ and thus describes only the initial region of the stress–strain curve, which does not vary significantly with use of coagulation treatment. However, μ_1_ is influenced by the non-linearity of the curve at higher strain ($\mu_{1}=\frac{2\,\mu_{0}}{\alpha_{1}}$). Importantly, the simulated stress–strain responses of each PVA/gelatin composition under compression, obtained using the mean values of the hyperelastic parameters of both constitutive models, compare well with the experimental test data. Despite the differences in constitutive parameters, both the first order Ogden and Yeoh models are appropriate for modelling the quasi-static behaviour of PVA/gelatin cryogel under compression.

Arterial wall stiffness is commonly probed using uniaxial and biaxial testing; for coronary arteries, the values range hugely from 0.9 kPa to 10 MPa (11). The experimental work of Holzapfel et al., which examined the layer-specific mechanical properties of human coronary arteries under tension (18), remains the basis of many computational studies where layer-specific wall properties are relevant to the simulation (35–37, 60–63). In all cases, a phenomenological approach is used to curve fit the experimental test data and extract the hyperelastic coefficients that best capture the “J”-shaped hyperelastic stress–strain curve. The optimal model is the one which provides the best fit across the required strain range. However, these cited studies differ in the material constitutive law used to fit the same experimental data. The rationale behind hyperelastic model choice is not always stated. Furthermore, the impact of hyperelastic model choice on the desired FE outputs is rarely investigated or compared. This study highlights the difference in transmural stress and strain, and subsequently compliance, using three isotropic strain energy density constitutive equations based on the same experimental data.

The results indicate that both circumferential (tensile) and radial (compressive) stress and strain primarily act upon the intimal layer of coronary arteries with non-atherosclerotic intimal thickening, regardless of hyperelastic model used. However, while the stiffness of each of the three layers is distinct when using the Ogden and polynomial models, the reduced polynomial model behaves like a bi-layered wall consisting of an intima and combined media–adventitia layer. These results not only have implications in the assessment of synthetic grafts, where arterial wall properties are needed as a benchmark for their performance, but for any FE model aiming to replicate the quasi-static biomechanics of the coronary artery wall. This is particularly relevant when modelling (or mimicking) mechanotransduction in the endothelium, as the forces generated by stress and strain within the wall play a concerted role in mediating healthy and disease-related endothelial cell behaviour (64). For instance, numerical analysis has shown that transmural pressure influences low-density lipoprotein accumulation across the intima and media, a key process in the progression of atherosclerotic plaques (65). Yet, the impact of the material model used to model these tissue layers was not discussed.

The stress–strain results of all three coronary artery models study align with the experimental data from which the hyperelastic parameters were fitted: at physiological pressures, the intima is largely responsible for the mechanical strength of the coronary artery (18). It displays the highest stiffness of all three tissue layers and bears the brunt of both circumferential and radial stresses. At the intima–media interface, σ_θ_ drops an order of magnitude while the gradient of σ_*r*_ decreases substantially. The contributions of the two layers to the mechanical properties of the wall must therefore be considered separately. This is in contrast to studies performed using porcine coronary artery data, where the intima and media are generally analysed as a combined intima–media layer (16, 17). These results reflect the limitations associated with using such bi-layered models (i.e., models comprised of intima–media and adventitia) as the basis to develop functional coronary artery grafts.

FE analysis has shown that, by keeping the composition of PVA/gelatin cryogel fixed across the intima and media and varying the stiffness of the adventitia, hyperelastic bi- or tri-layered synthetic grafts of variable mechanical stiffness can be constructed. Qualitatively, PG-4 (where the same cryogel composition is used as both the media and adventitia mimic) captures the behaviour of the bi-layered reduced polynomial arterial model, while PG-1, -2 and -3 resemble the tri-layered Ogden and polynomial arterial models. Mimicking the additional stiffness provided by the adventitia in the tri-layered models is achieved by choosing a cryogel composition where σ_θ,*adventitia*_ > σ_θ,*media*_; this has been accomplished by increasing the *M*_*w*_ of PVA and/or employing polymer coagulation to increase the crystalline domain of PVA. Crucially, increasing the stiffness of the adventitia mimicking material influences the transmural distribution of stress and strain in the graft. σ_θ_ distribution is reduced in the intima and increased in the adventitia. The compliance of the graft also decreases. While this study has focused on PVA/gelatin grafts, the importance of layer stiffness is translatable to other hyperelastic VMMs and should be considered when designing any multi-layered graft.

Quantitatively, several differences are observed in the stress–strain behaviour of the synthetic grafts and arterial tissue. Despite strain increasing linearly between diastole and systole in both the artery and synthetic models, the coronary artery exhibits significant strain stiffening at pressures below diastole. PVA/gelatin, however, does not; at systolic pressure, the ϵ_θ_ and ϵ_*r*_ of PVA/gelatin grafts reached up to double that in native tissue. Furthermore, the relative error between graft and artery was highly sensitive to the constitutive models used to describe them. The compliance of all four synthetic grafts at physiological pressure was notably higher than the compliance of each coronary artery model. Compliance values are lacking in the majority of multi-layered artery graft literature, despite it being a critical parameter in determining graft success (19). From the perspective of shear stress exerted at the blood–endothelium interface, mismatched wall compliance changes the cross-sectional area of the vessel and thus flow through the vessel, particularly at anastomoses. Given the impact that manufacturing parameters have on the mechanical properties of PVA/gelatin cryogel, designing stiffer PVA/gelatin compositions with enhanced strain-stiffening behaviour could reduce the compliance and better fit the requirements of synthetic coronary artery grafts.

There are certain limitations in the study that should be addressed. Firstly, viscoelasticity and anisotropy were not incorporated into any of the constitutive material models used. As the frequency and stress relaxation responses of PVA/gelatin were not measured in this study, viscoelastic effects were neglected. The quasi-static nature of this study renders this an applicable simplification. If future work seeks to investigate dynamic loads, or the time-dependent stress response of artery and graft, viscoelasticity would need to be represented in the constitutive material model. Arteries display direction-dependent stress–stretch responses (18). As longitudinal motion was constrained in this study, isotropic hyperelastic coefficients fitted to circumferential stress–stretch data were applicable and hence sourced from the literature (Table 2). The casting method used to manufacture PVA/gelatin cryogels produced isotropic samples. In future, anisotropy may be engineered into PVA/gelatin cryogels by introducing a freezing gradient during the initial freezing step (39, 66) or through cryogenic 3D printing (29).

The effects of residual stresses (axially, radially and circumferentially) were not incorporated into the coronary artery models; that is, when free of external load, the models were assumed to experience a zero-stress state. Residual stresses affect the uniformity of transmural stress in arteries (67), and the magnitude of σ_θ_ at the luminal surface has been shown to reduce significantly in their presence (68). Consequently, the disparity in σ_θ_ at the surface of PVA/gelatin grafts and the coronary artery may be less significant than reported in this study. While future efforts should be made to include the effects of residual stress and strain, the qualitative trends reported here – the relative magnitudes of σ_θ_ and σ_*r*_, the discontinuity of σ_θ_ at the media–adventitia interface and the off-loading of stress from the intima to the adventitia – match those reported when residual stresses are considered (68). In addition, the perivascular pressure (exemplified here by embedding the artery and graft in a fixed block of surrounding connective tissue) will influence stress and strain distribution through the vessel wall. The properties and boundary conditions associated with this surrounding tissue should thus be explored further in future work.

To allow relative motion between the heterogeneous boundaries of the simulated coronary artery wall and PVA/gelatin grafts, the interfacing surfaces of the mesh were allocated hard penalty contact constraints and a friction coefficient. For this quasi-static study of the transmural biomechanics of a laminated artery and graft, this is an applicable assumption. However, it should be noted that further research involving dynamic loading conditions requires further research into how to most accurately define the behaviour of these interfacing surfaces. There is negligible literature defining this behaviour. The friction coefficient used between the layers of the artery wall, for example, was taken from the literature (45). The authors note that a friction coefficient of 0.2 was assumed “for all contact pairs”, though they do not explicitly identify which surfaces this applies to. The interactions between two PVA surfaces are even less defined and will be influenced by the manufacturing protocol. Crolla et al. demonstrated that 3D printed PVA cryogel fails at the interfacing boundaries between the printed filaments when tested under tension (29), suggesting that simply tying the surfaces together and constraining translational and rotational motion is unsuitable for representing the contact behaviour. As the model complexity is increased to incorporate the effects of wall viscoelasticity and complex geometries, research into the interfacing behaviour must be undertaken.

## Conclusion

The transmural biomechanics of multi-layered coronary artery grafts is a largely unexplored area of cardiovascular research, despite the important role of wall stress and strain distribution on vascular (dys)function. The hyperelastic constitutive parameters for PVA/gelatin cryogel, a versatile biomaterial capable of replicating the biomechanics of soft tissue, were first extracted from uniaxial compression data. Then, using experimentally-informed finite element models, the quasi-static responses of four PVA/gelatin graft designs were evaluated relative to the coronary artery wall. In all cases, stress and strain distribution were heavily influenced by the constitutive model used to capture the hyperelastic response of each material, despite being fitted to the same experimental data. Bi- and tri-layered grafts were obtained by varying the stiffness of the adventitia mimic relative to the stiffness of the media mimic: increasing the adventitial stiffness reduced the maximal stresses observed at the luminal surface of the graft and decreased graft compliance. Importantly, the qualitative trends of stress and strain distribution matched those observed in the coronary artery.

This article presents a new method for designing multi-layered hydrogel grafts that synergises experimental data with computational simulations to enhance our current understanding of the transmural biomechanics of synthetic grafts under physiological loading. The results highlight the impact of hyperelastic model choice in replicating arterial mechanical behaviour, a finding that has significance across the wider field of finite element analysis in cardiovascular mechanics. Moreover, the results highlight the inadequacy of homogeneous constructs to replicate the biomechanics of the native wall and emphasise the need for biomimetic, multi-layered graft designs. Identifying the design parameters that affect the transmural biomechanics of synthetic grafts will facilitate better understanding of graft performance. Thus, future work should look at validating the results of the FE model by seeding the multi-layer graft designs proposed here and assessing the impact of cyclic stretch on EC behaviour. While FE analysis shows that the grafts were considerably more compliant than the native artery wall, the proposed method is applicable to the development of other hyperelastic VMMs for cardiovascular tissue engineering.

## Data Availability Statement

The original contributions presented in this study are included in the article/Supplementary Material, further inquiries can be directed to the corresponding author.

## Ethics Statement

Ethical review and approval was not required for this study in accordance with the local legislation and institutional requirements.

## Author Contributions

KF contributed to the conception, design and implementation of the research, to the analysis of the results, and to writing the original draft of the manuscript. NG supervised and contributed to the conception and design of the research. MB and AI supervised the research. LT-S supervised and contributed to the conception, design and implementation of the research. All authors contributed to manuscript revision, read, and approved the submitted version.

## Conflict of Interest

The authors declare that the research was conducted in the absence of any commercial or financial relationships that could be construed as a potential conflict of interest.

## Publisher’s Note

All claims expressed in this article are solely those of the authors and do not necessarily represent those of their affiliated organizations, or those of the publisher, the editors and the reviewers. Any product that may be evaluated in this article, or claim that may be made by its manufacturer, is not guaranteed or endorsed by the publisher.
