# Supplementary material for: Design and Simulation of the Biomechanics of Multi-Layered Composite Poly(Vinyl Alcohol) Coronary Artery Grafts
Source: Front Cardiovasc Med. 2022 Jun 24;9:883179. doi: 10.3389/fcvm.2022.883179 (PMC9272978; doi:10.3389/fcvm.2022.883179)
Supplement: Supplementary file 1 [file Data_Sheet_1.PDF]

Supplementary Material

1 Supplementary Figures and Tables

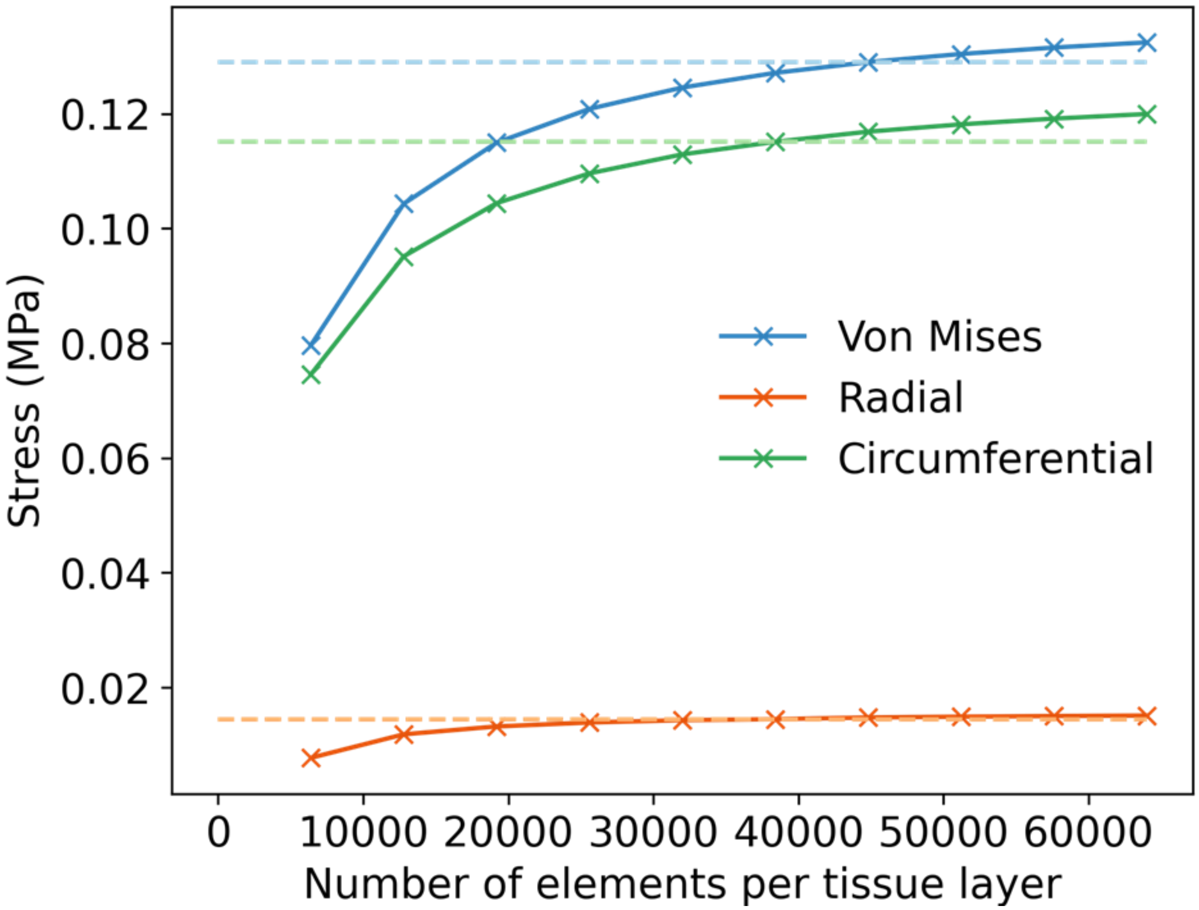

Figure S1. Mesh convergence study performed using 3rd order Ogden coronary artery coefficients at 120 mmHg pressure. Convergence was achieved when the stress increased less than 2% relative to the previous mesh size (dashed lines).

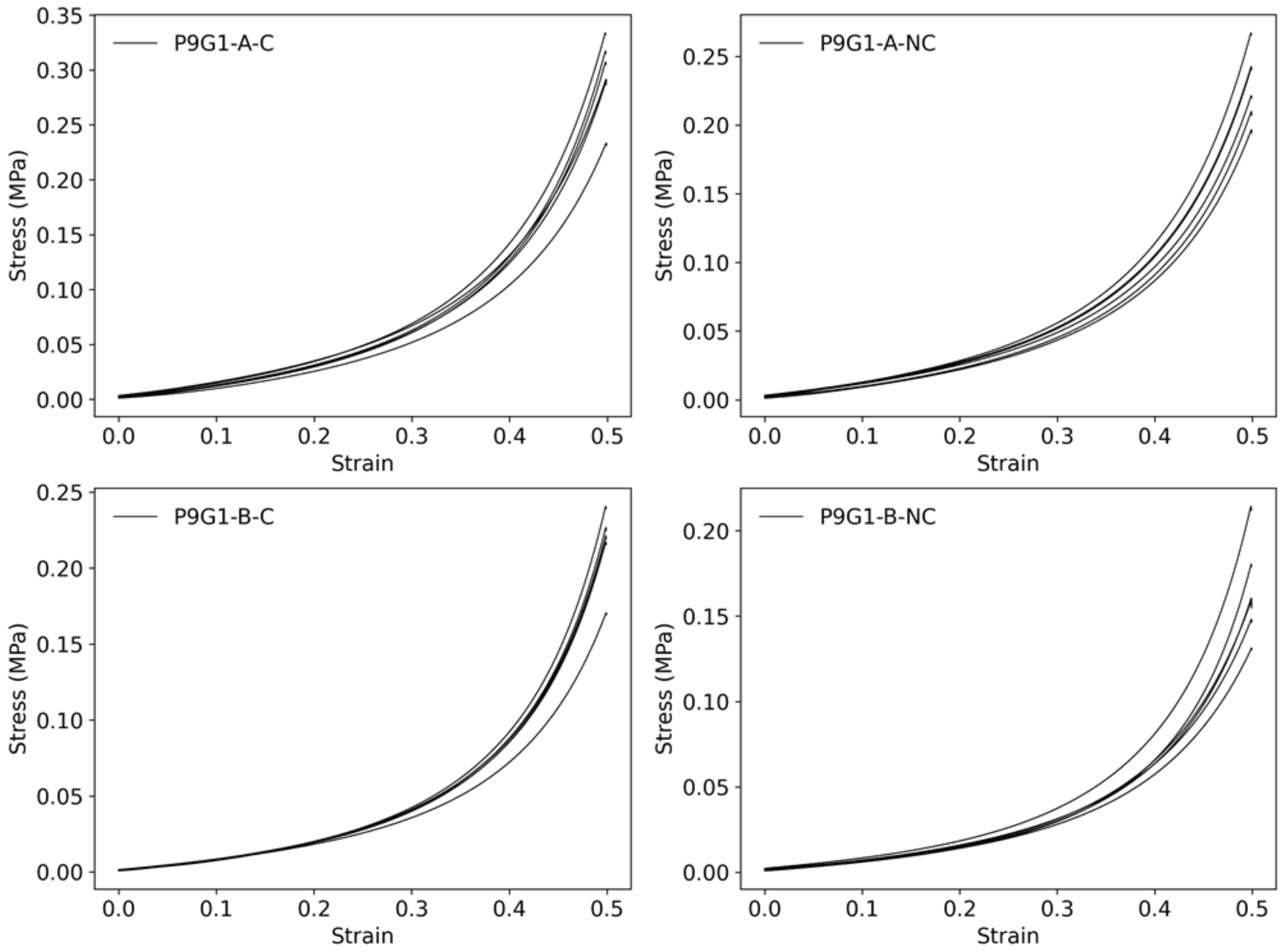

Figure S2. Individual stress–strain curves (test data) for P_9G_1-A-C, P_9G_1-A-NC; P_9G_1-B-C and P_9G_1-B-NC.

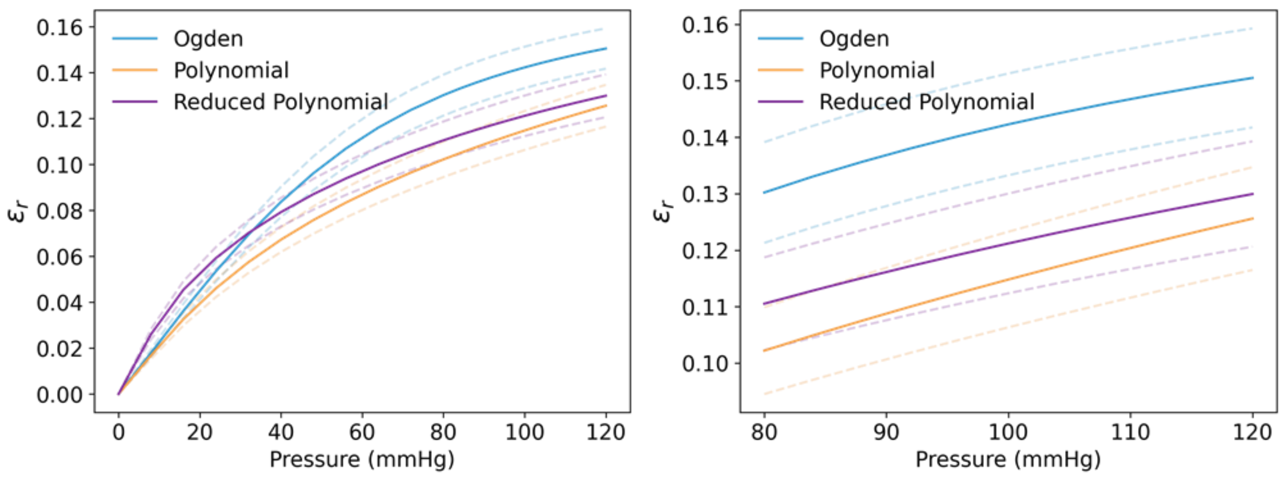

Figure S3. The mean ϵ_r of the coronary intima between 0–120 and 80–120 mmHg pressure; dashed lines show the S.D. of each model.

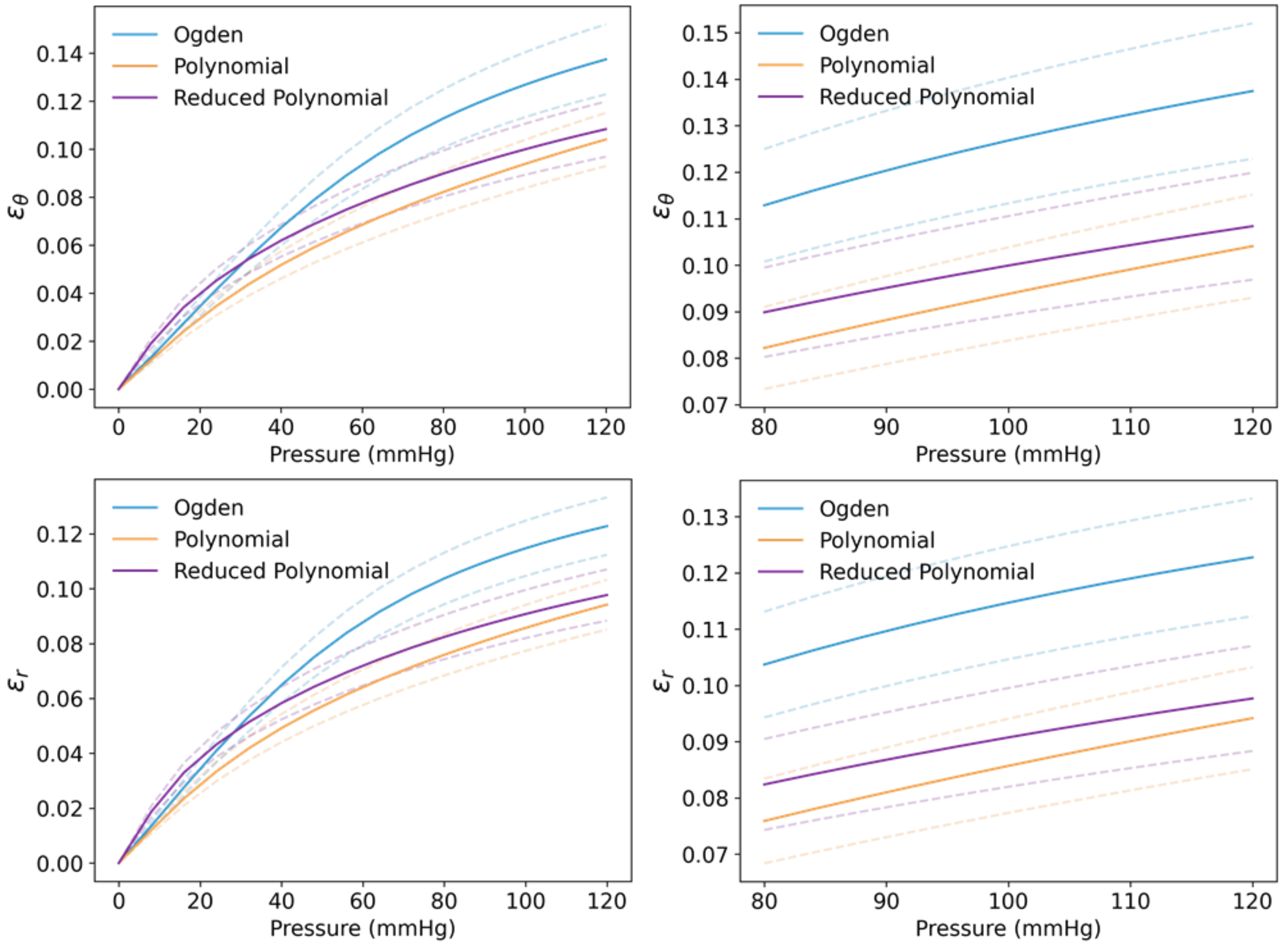

Figure S4. The mean ϵ_θ (top row) and ϵ_r (bottom row) of the coronary media between 0–120 and 80–120 mmHg pressure; dashed lines show the S.D. of each model.

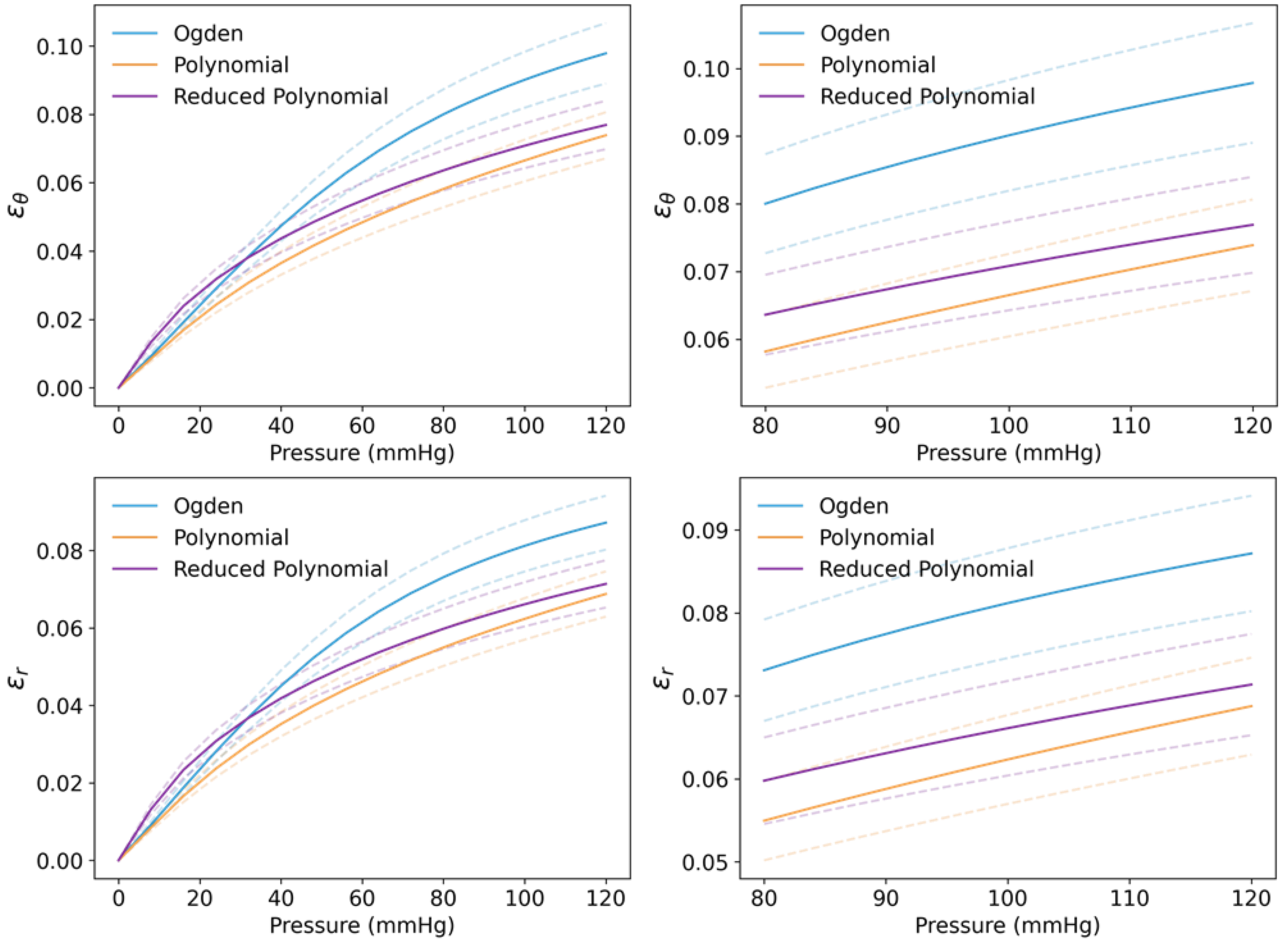

Figure S5. The mean ϵ_θ (top row) and ϵ_r (bottom row) of the coronary adventitia between 0–120 and 80–120 mmHg pressure; dashed lines show the S.D. of each model.

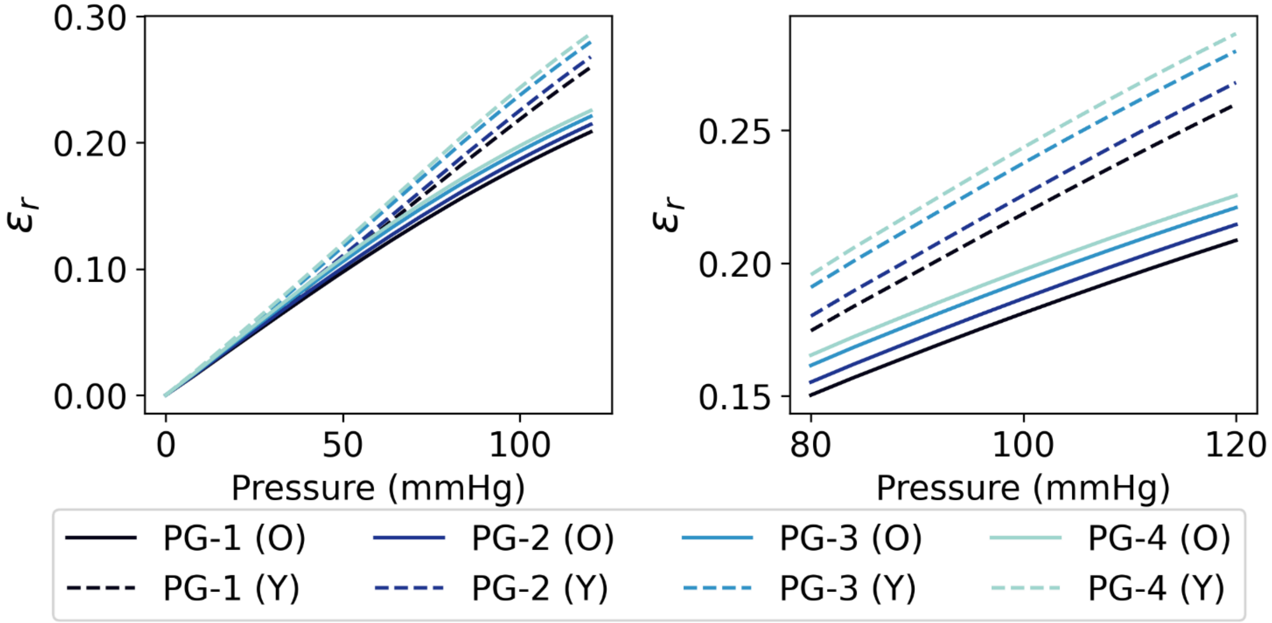

Figure S6. The mean ϵ_r of PVA/gelatin graft intima between 0–120 and 80–120 mmHg pressure; dashed lines show the S.D. of each model (error bars have been omitted for clarity).

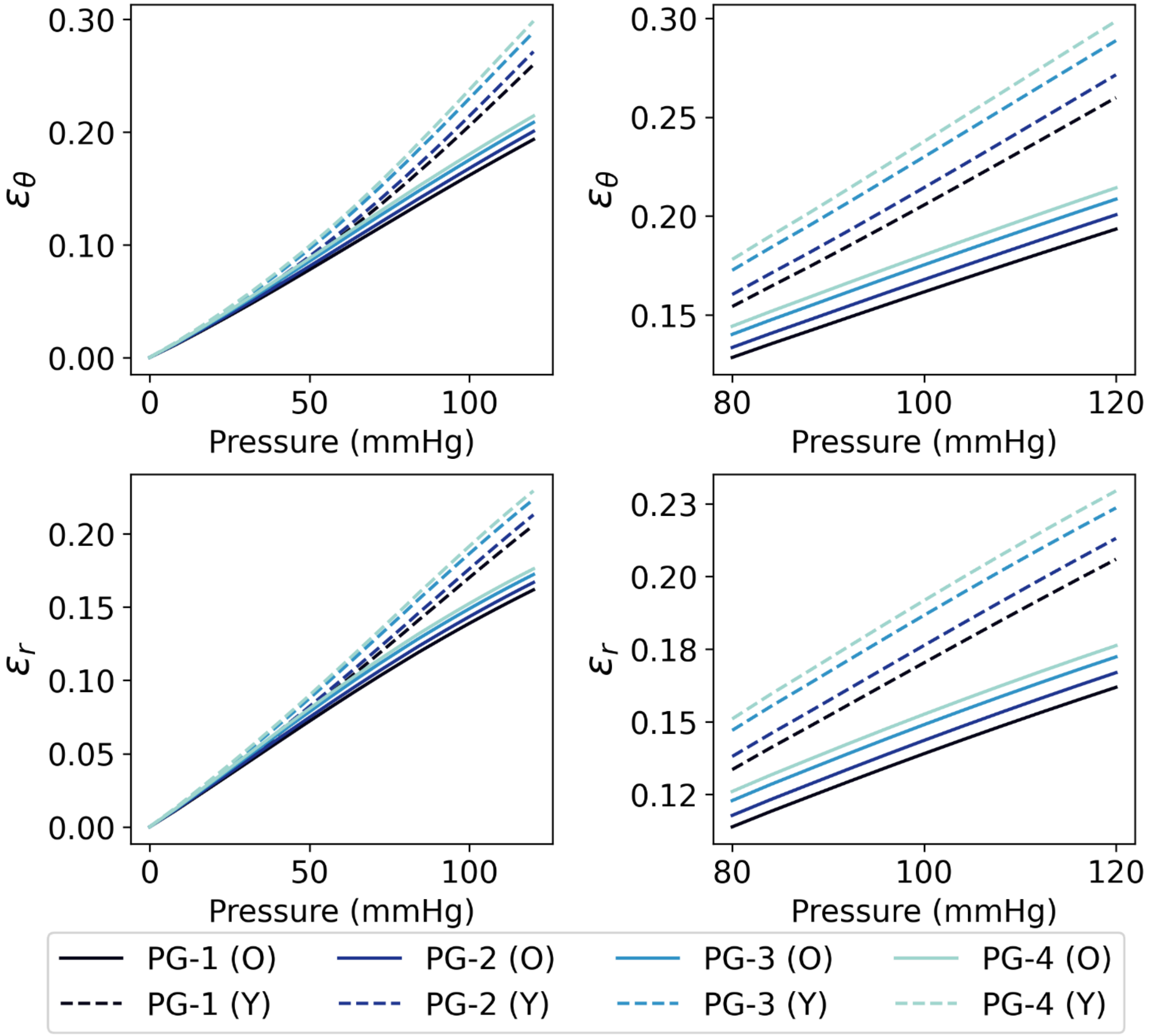

Figure S7. The mean ϵ_θ (top row) and ϵ_r (bottom row) of PVA/gelatin graft media between 0–120 and 80–120 mmHg pressure (error bars have been omitted for clarity).

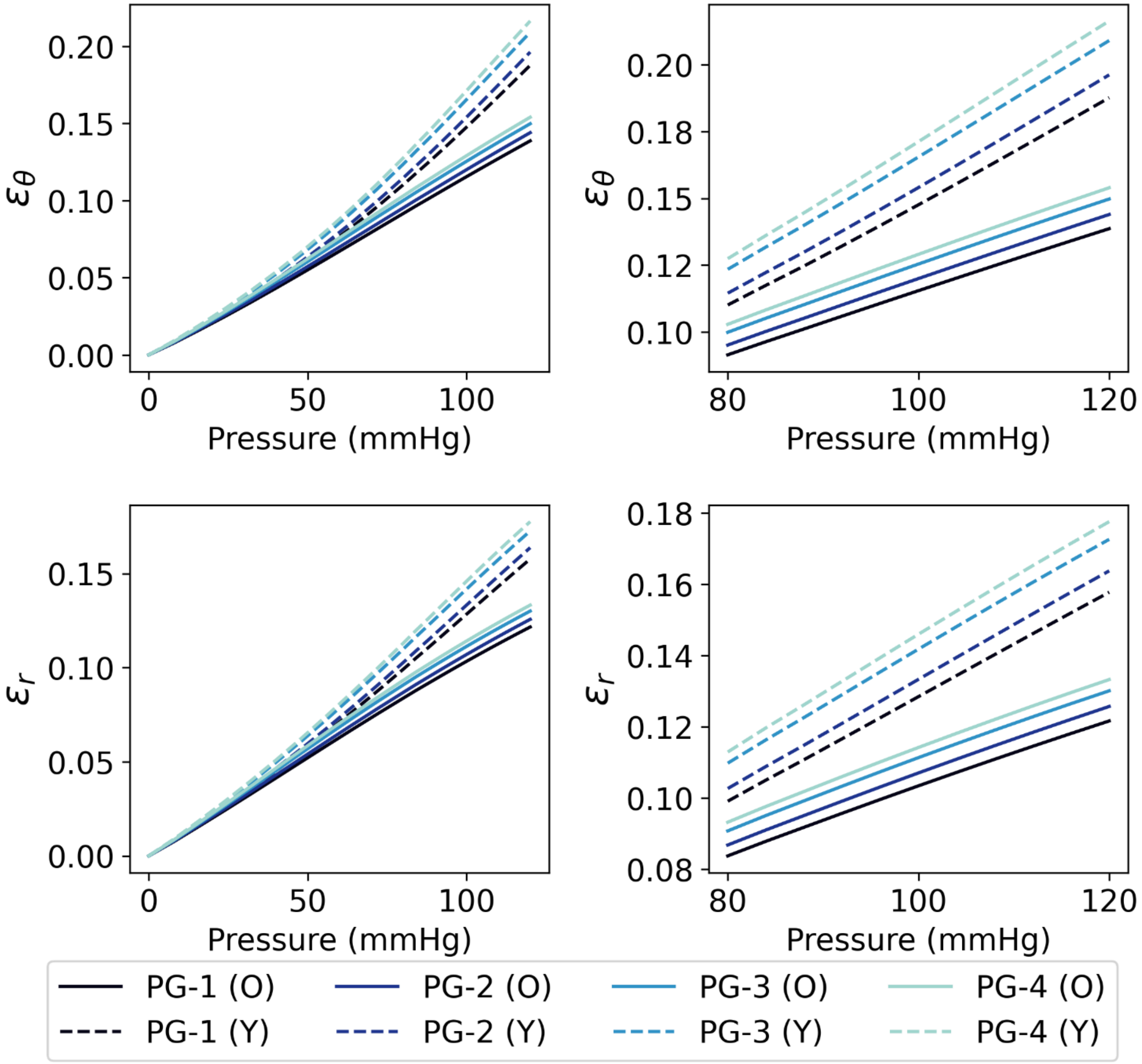

Figure S8. The mean ϵ_θ (top row) and ϵ_r (bottom row) of PVA/gelatin graft adventitia between 0–120 and 80–120 mmHg pressure (error bars have been omitted for clarity).

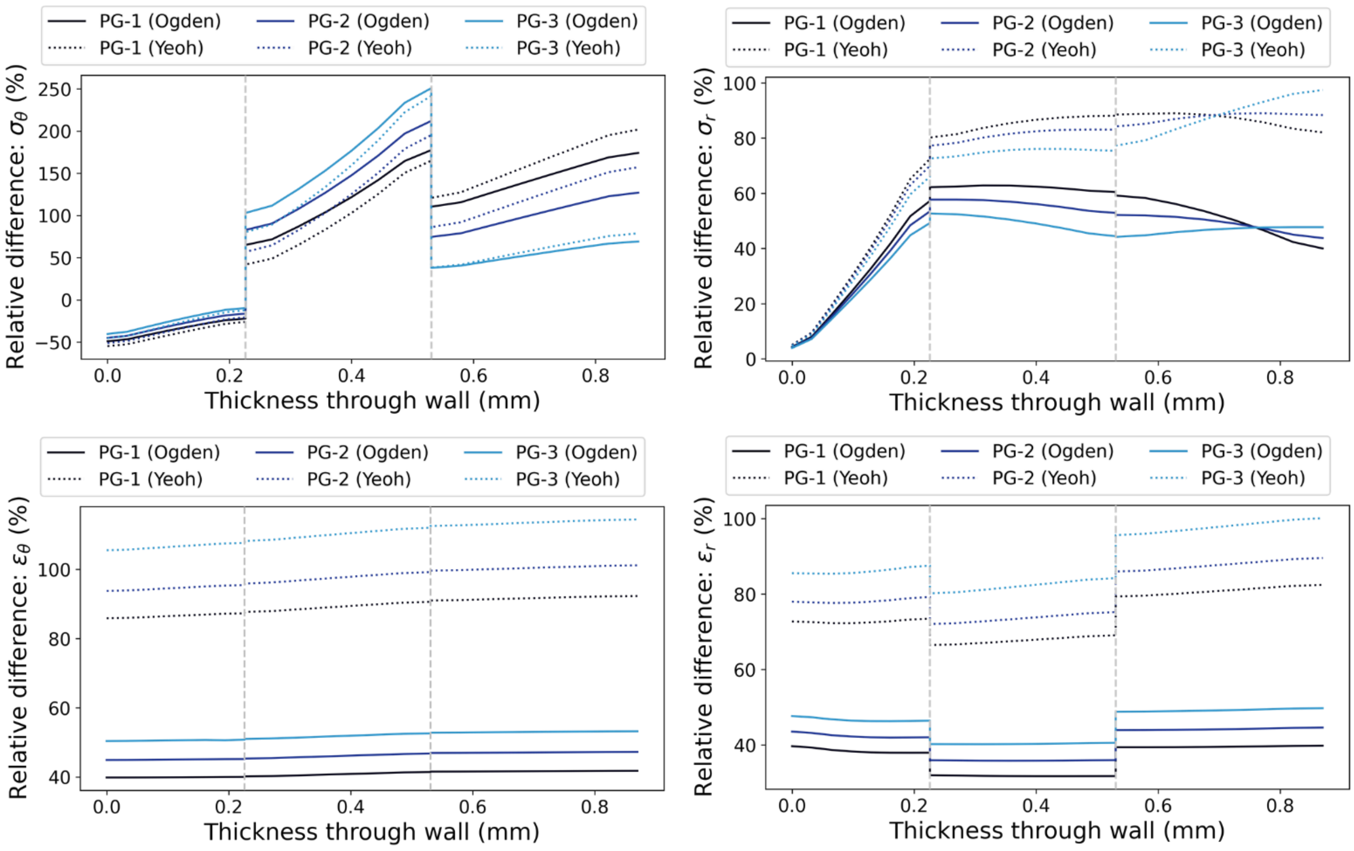

Figure S9. The relative difference (%) in σ_θ, σ_r, ϵ_θ and ϵ_r between tri-layered PVA/gelatin grafts and the Ogden coronary artery model. The solid and dotted lines represent PVA/gelatin grafts modelled using Ogden and Yeoh hyperelastic parameters, respectively. Grey dashed lines represent the intima–media and media–adventitia interfaces.

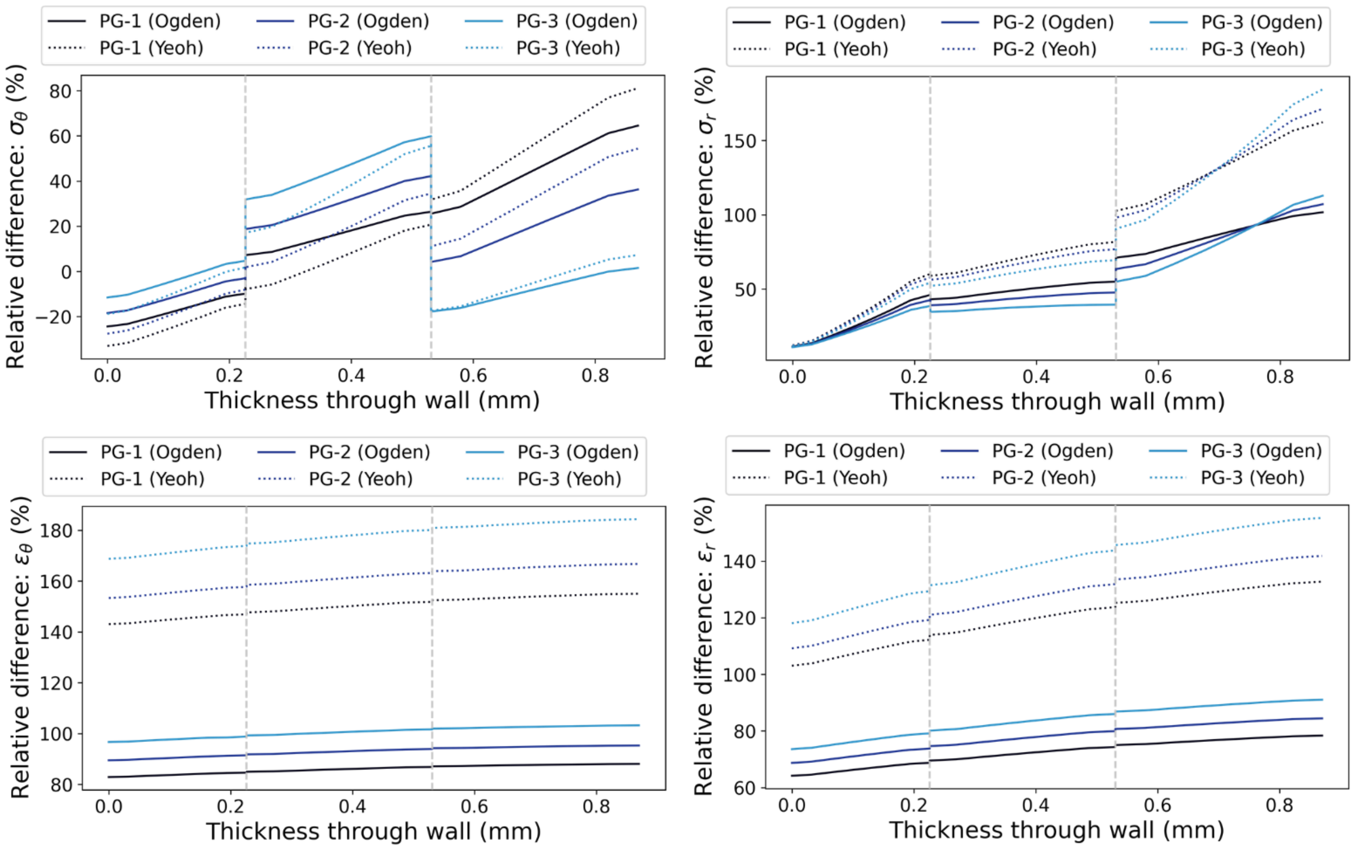

Figure S10. The relative difference (%) in σ_θ, σ_r, ϵ_θ and ϵ_r between tri-layered PVA/gelatin grafts and the Polynomial coronary artery model. The solid and dotted lines represent PVA/gelatin grafts modelled using Ogden and Yeoh hyperelastic parameters, respectively. Grey dashed lines represent the intima–media and media–adventitia interfaces.

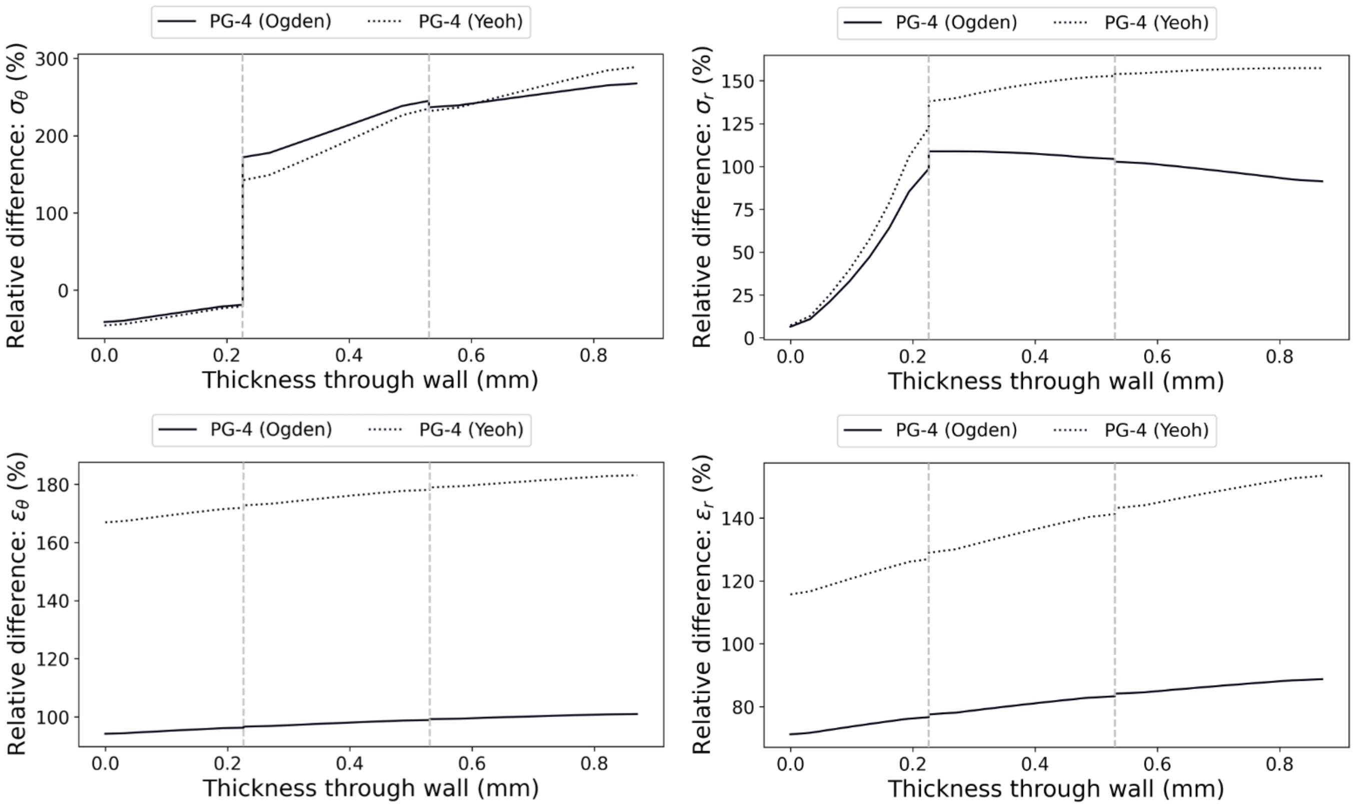

Figure S11. The relative difference (%) in σ_θ, σ_r, ϵ_θ and ϵ_r between the bi-layered PVA/gelatin graft PG-4 and the reduced polynomial coronary artery model. The solid and dotted lines represent PVA/gelatin grafts modelled using Ogden and Yeoh hyperelastic parameters, respectively. Grey dashed lines represent the intima–media and media–adventitia interfaces.

Table S1. First order Ogden parameters and r^2 values for individual PVA/gelatin samples. For P_9G_1-B-C, samples marked by ** yielded $\alpha_1 < 0$ were excluded to allow averaging of α_1. For P_9G_1-A-C, P_9G_1-A-NC and P_9G_1-B-NC, samples marked by ** were excluded to keep the sample size consistent between compositions (n=6). The n=6 mean values were the hyperelastic constants used in subsequent FE models.

**P_9G_1-A-C**			
**Sample**	**μ_1 (MPa)**	**α_1**	**r^2**
**1**	0.0448	7.330	0.9993
**2**	0.0433	7.169	0.9999
**3**	0.0502	7.065	0.9995
**4**	0.0416	7.672	0.9998
**5**	0.0516	6.122	0.9988
**6**	0.0352	7.337	0.9993
**7** **	0.0460	6.718	0.9998
**8** **	0.0475	6.461	0.9984
**Average (n=8)**	0.0450 $\pm$ 0.0052	6.984 $\pm$ 0.513	0.9994 $\pm$ 0.0004
**Average (n=6)**	0.0444 $\pm$ 0.0060	7.116 $\pm$ 0.528	0.9994 $\pm$ 0.0004
**P_9G_1-A-NC**			
**Sample**	**μ_1 (MPa)**	**α_1**	**r^2**
**1**	0.0409	6.261	0.9978

**2**	0.0417	6.782	0.9991
**3**	0.0335	6.725	0.9995
**4**	0.0379	6.207	0.9981
**5**	0.0313	6.854	0.9999
**6**	0.0394	6.488	0.9986
**7****	0.0370	6.807	0.9991
**8****	0.0355	6.968	0.9996
**Average (n=8)**	0.0371 $\pm$ 0.0036	6.636 $\pm$ 0.283	0.9989 $\pm$ 0.0008
**Average (n=6)**	0.0374 $\pm$ 0.0042	6.553 $\pm$ 0.276	0.9988 $\pm$ 0.0008
**P_9G_1-B-C**			
**Sample**	**μ_1 (MPa)**	**α_1**	**r^2**
**1**	0.0260	6.923	0.9995
**2**	0.0274	7.903	0.9995
**3**	0.0288	7.516	0.9989
**4**	0.0279	8.308	0.9997
**5****	0.0167	−3.470	0.9980
**6****	0.0176	−2.944	0.9946

**7**	0.0271	8.120	0.9997
**8**	0.0285	7.819	0.9992
**Average (n=8)**	0.0250 ± 0.0049	5.022 ± 5.098	0.9986 ± 0.0017
**Average (n=6)**	0.0276 ± 0.0010	7.765 ± 0.493	0.9994 ± 0.0003
	**P_9G_1-B-NC**		
**Sample**	**μ_1 (MPa)**	**α_1**	**r^2**
**1**	0.0229	7.172	0.9992
**2**	0.0235	6.680	0.9990
**3**	0.0201	8.252	0.9987
**4**	0.0209	6.760	0.9996
**5**	0.0216	7.421	0.9988
**6**	0.0271	7.580	0.9973
**7****	0.0198	7.225	0.9995
**8****	0.0197	7.385	0.9999
**Average (n=8)**	0.0220 ± 0.0025	7.309 ± 0.493	0.9990 ± 0.0008
**Average (n=6)**	0.0227 ± 0.0025	7.311 ± 0.581	0.9988 ± 0.0008

Table S2. Individual Mooney-Rivlin parameters fitted to PVA/gelatin test data. Coefficients fulfilling Drucker's stability criterion are deemed stable, while those where that failed are deemed unstable.

P$_9$G$_1$-A-C				
**Sample**	**C_{10} (MPa)**	**C_{01} (MPa)**	**r^2**	**Stable?**
**1**	−0.0017	0.019	0.9845	No
**2**	−0.0012	0.018	0.9897	No
**3**	0.0003	0.019	0.9863	Yes
**4**	−0.0048	0.020	0.9879	No
**5**	0.0087	0.013	0.9863	Yes
**6**	−0.0026	0.016	0.9946	No
**7**	0.0027	0.016	0.9896	Yes
**8**	0.0056	0.014	0.9837	Yes
**P$_9$G$_1$-A-NC**				
**Sample**	**C_{10} (MPa)**	**C_{01} (MPa)**	**r^2**	**Stable?**
**1**	0.0064	0.011	0.9821	Yes
**2**	0.0027	0.014	0.9849	Yes
**3**	0.0020	0.011	0.9875	Yes
**4**	0.0061	0.010	0.9835	Yes
**5**	0.0010	0.011	0.9909	Yes

**6**	0.0045	0.012	0.9840	Yes
**7**	0.0021	0.013	0.9850	Yes
**8**	0.0007	0.013	0.9869	Yes
**P_9G_1-B-C**				
**Sample**	**C_{10} (MPa)**	**C_{01} (MPa)**	**r^2**	**Stable?**
**1**	0.0009	0.009	0.9863	Yes
**2**	−0.0041	0.014	0.9827	No
**3**	−0.0020	0.013	0.9813	No
**4**	−0.0067	0.017	0.9834	No
**5**	−0.0103	0.018	0.9890	No
**6**	−0.0058	0.014	0.9878	No
**7**	−0.0060	0.016	0.9869	No
**8**	−0.0037	0.014	0.9816	No
**P_9G_1-B-NC**				
**Sample**	**C_{10} (MPa)**	**C_{01} (MPa)**	**r^2**	**Stable?**
**1**	−0.005	0.009	0.9838	No
**2**	0.0018	0.008	0.9851	Yes

**3**	−0.0042	0.011	0.9765	No
**4**	0.0012	0.007	0.9877	Yes
**5**	−0.0011	0.009	0.9810	No
**6**	−0.0019	0.012	0.9747	No
**7**	−0.0005	0.008	0.9853	No
**8**	−0.0012	0.009	0.9883	No

Table S3. Constitutive parameters of stable hyperelastic models fitted to PVA/gelatin compression data and densities of all PVA/gelatin cryogel compositions (mean $\pm$ S.D.).

**First Order Ogden Constitutive Parameters**				
	**P9G1-A-C**	**P9G1-A-NC**	**P9G1-B-C**	**P9G1-B-NC**
$\boldsymbol{\mu_1}$ **(MPa)**	0.0444 $\pm$ 0.0060	0.0374 $\pm$ 0.0042	0.0276 $\pm$ 0.0010	0.0227 $\pm$ 0.0025
$\boldsymbol{\alpha_1}$	7.116 $\pm$ 0.528	6.553 $\pm$ 0.276	7.765 $\pm$ 0.493	7.311 $\pm$ 0.581
$\boldsymbol{\mu_0}$ **(MPa)**	0.157 $\pm$ 0.016	0.122 $\pm$ 0.012	0.107 $\pm$ 0.009	0.083 $\pm$ 0.011
$\boldsymbol{r^2}$	0.9994 $\pm$ 0.0004	0.9988 $\pm$ 0.0008	0.9994 $\pm$ 0.0003	0.9988 $\pm$ 0.0008
**Neo-Hookean Constitutive Parameters**				
	**P9G1-A-C**	**P9G1-A-NC**	**P9G1-B-C**	**P9G1-B-NC**
$\boldsymbol{C_{10}}$ **(MPa)**	0.0239 $\pm$ 0.0029	0.0198 $\pm$ 0.0021	0.0152 $\pm$ 0.0007	0.0123 $\pm$ 0.0014
$\boldsymbol{\mu_0}$ **(MPa)**	0.0479 $\pm$ 0.0059	0.0396 $\pm$ 0.0043	0.0304 $\pm$ 0.0014	0.0247 $\pm$ 0.0027
$\boldsymbol{r^2}$	0.9483 $\pm$ 0.0083	0.9505 $\pm$ 0.0032	0.9319 $\pm$ 0.0080	0.9329 $\pm$ 0.0151
**Second Order Reduced Polynomial Constitutive Parameters**				
	**P9G1-A-C**	**P9G1-A-NC**	**P9G1-B-C**	**P9G1-B-NC**
$\boldsymbol{C_{10}}$ **(MPa)**	0.0190 $\pm$ 0.0028	0.0162 $\pm$ 0.0019	0.0116 $\pm$ 0.0004	0.0096 $\pm$ 0.0011
$\boldsymbol{C_{20}}$ **(MPa)**	0.0076 $\pm$ 0.0012	0.0050 $\pm$ 0.0006	0.0060 $\pm$ 0.0011	0.0042 $\pm$ 0.0009
$\boldsymbol{\mu_0}$ **(MPa)**	0.0380 $\pm$ 0.0056	0.0325 $\pm$ 0.0038	0.0231 $\pm$ 0.0008	0.0176 $\pm$ 0.0044
$\boldsymbol{r^2}$	0.9951 $\pm$ 0.0022	0.9929 $\pm$ 0.0023	0.9929 $\pm$ 0.0014	0.9912 $\pm$ 0.0030
**Yeoh Constitutive Parameters**				
	**P9G1-A-C**	**P9G1-A-NC**	**P9G1-B-C**	**P9G1-B-NC**

C_{10} (MPa)	0.0205 ± 0.0035	0.0180 ± 0.0024	0.0126 ± 0.0006	0.0108 ± 0.0014
C_{20} (MPa)	0.0015 ± 0.0028	-0.0015 ± 0.0019	0.0015 ± 0.0014	-0.0007 ± 0.0007
C_{30} (MPa)	0.0042 ± 0.0017	0.0044 ± 0.0012	0.0032 ± 0.0006	0.0033 ± 0.0009
μ_0 (MPa)	0.0410 ± 0.0070	0.0360 ± 0.0049	0.0252 ± 0.0012	0.0217 ± 0.0027
r^2	0.9994 ± 0.0008	0.9984 ± 0.0009	0.9997 ± 0.0002	0.9985 ± 0.0007
**Fifth Order Reduced Polynomial Constitutive Parameters**				
	**P9G1-A-C**	**P9G1-A-NC**	**P9G1-B-C**	**P9G1-B-NC**
C_{10} (MPa)	0.0218 ± 0.0044	0.0198 ± 0.0031	0.0133 ± 0.0008	0.0121 ± 0.0017
C_{20} (MPa)	-0.0123 ± 0.0110	-0.0192 ± 0.0085	-0.0064 ± 0.0035	-0.0133 ± 0.0038
C_{30} (MPa)	0.0402 ± 0.0212	0.0484 ± 0.0168	0.0251 ± 0.0068	0.0355 ± 0.0083
C_{40} (MPa)	-0.0343 ± 0.0174	-0.0407 ± 0.0140	-0.0217 ± 0.0058	-0.0303 ± 0.0071
C_{50} (MPa)	0.0110 ± 0.0052	0.0126 ± 0.0043	0.0072 ± 0.0019	0.0095 ± 0.0023
μ_0 (MPa)	0.0435 ± 0.0087	0.0396 ± 0.0062	0.0265 ± 0.0017	0.0241 ± 0.0034
r^2	0.9992 ± 0.0006	0.9985 ± 0.0007	0.9993 ± 0.0003	0.9981 ± 0.0005

Table S4. Correlation matrices showing the effect of M_w PVA and coagulation treatment on μ_0 of PVA/gelatin cryogel. NS = not statistically significant ($p > 0.05$); * = $p \leq 0.05$; ** = $p \leq 0.01$; *** = $p \leq 0.001$.

		**1st Order Ogden**		
	**P_9G_1-A-C**	**P_9G_1-A-NC**	**P_9G_1-B-C**	**P_9G_1-B-NC**
**P_9G_1-A-C**		***	***	***
**P_9G_1-A-NC**	***		*	***
**P_9G_1-B-C**	***	*		**
**P_9G_1-B-NC**	***	***	**	
		**Neo-Hookean**		
	**P_9G_1-A-C**	**P_9G_1-A-NC**	**P_9G_1-B-C**	**P_9G_1-B-NC**
**P_9G_1-A-C**		**	***	***
**P_9G_1-A-NC**	**		**	***
**P_9G_1-B-C**	***	**		*
**P_9G_1-B-NC**	***	***	*	
		**Second Order Reduced Polynomial**		
	**P_9G_1-A-C**	**P_9G_1-A-NC**	**P_9G_1-B-C**	**P_9G_1-B-NC**
**P_9G_1-A-C**		*	***	***
**P_9G_1-A-NC**	*		**	***
**P_9G_1-B-C**	***	**		NS
**P_9G_1-B-NC**	***	***	NS	
		**Yeoh**		
	**P_9G_1-A-C**	**P_9G_1-A-NC**	**P_9G_1-B-C**	**P_9G_1-B-NC**
**P_9G_1-A-C**		NS	***	***
**P_9G_1-A-NC**	NS		**	***
**P_9G_1-B-C**	***	**		NS
**P_9G_1-B-NC**	***	***	NS	
		**Fifth Order Reduced Polynomial**		

	**P_9G_1-A-C**	**P_9G_1-A-NC**	**P_9G_1-B-C**	**P_9G_1-B-NC**
**P_9G_1-A-C**		NS	***	***
**P_9G_1-A-NC**	NS		**	***
**P_9G_1-B-C**	***	**		NS
**P_9G_1-B-NC**	***	***	NS	
